# European Psychiatric Association guidance on treatment of cognitive impairment in schizophrenia

**DOI:** 10.1192/j.eurpsy.2022.2315

**Published:** 2022-09-05

**Authors:** Antonio Vita, Wolfgang Gaebel, Armida Mucci, Gabriele Sachs, Stefano Barlati, Giulia Maria Giordano, Gabriele Nibbio, Merete Nordentoft, Til Wykes, Silvana Galderisi

**Affiliations:** 1 Department of Clinical and Experimental Sciences, University of Brescia, Brescia, Italy; 2 Department of Mental Health and Addiction Services, Spedali Civili Hospital, Brescia, Italy; 3 WHO Collaborating Centre on Quality Assurance and Empowerment in Mental Health DEU-131, LVR-Klinikum Düsseldorf, Düsseldorf, Germany; 4 Department of Psychiatry and Psychotherapy, Heinrich-Heine-University, Düsseldorf, Germany; 5 University of Campania “Luigi Vanvitelli”, Naples, Italy; 6 University of Vienna, Wien, Austria; 7 Department of Molecular and Translational Medicine, University of Brescia, Brescia, Italy; 8 CORE – Copenhagen Research Centre for Mental Health, Copenhagen University Hospital, Copenhagen, Denmark; 9 Department of Clinical Medicine, University of Copenhagen, Copenhagen, Denmark; 10 Department of Psychology, Institute of Psychiatry, Psychology and Neuroscience, King’s College London, London, United Kingdom; 11 South London and Maudsley NHS Foundation Trust, Maudsley Hospital, London, United Kingdom

**Keywords:** Cognitive enhancement, cognitive functioning, cognitive remediation, evidence-based, systematic review

## Abstract

**Background:**

Although cognitive impairment is a core symptom of schizophrenia related to poorer outcomes in different functional domains, it still remains a major therapeutic challenge. To date, no comprehensive treatment guidelines for cognitive impairment in schizophrenia are implemented.

**Methods:**

The aim of the present guidance paper is to provide a comprehensive meta-review of the current available evidence-based treatments for cognitive impairment in schizophrenia. The guidance is structured into three sections: pharmacological treatment, psychosocial interventions, and somatic treatments.

**Results:**

Based on the reviewed evidence, this European Psychiatric Association guidance recommends an appropriate pharmacological management as a fundamental starting point in the treatment of cognitive impairment in schizophrenia. In particular, second-generation antipsychotics are recommended for their favorable cognitive profile compared to first-generation antipsychotics, although no clear superiority of a single second-generation antipsychotic has currently been found. Anticholinergic and benzodiazepine burdens should be kept to a minimum, considering the negative impact on cognitive functioning. Among psychosocial interventions, cognitive remediation and physical exercise are recommended for the treatment of cognitive impairment in schizophrenia. Noninvasive brain stimulation techniques could be taken into account as add-on therapy.

**Conclusions:**

Overall, there is definitive progress in the field, but further research is needed to develop specific treatments for cognitive impairment in schizophrenia. The dissemination of this guidance paper may promote the development of shared guidelines concerning the treatment of cognitive functions in schizophrenia, with the purpose to improve the quality of care and to achieve recovery in this population.

## Introduction

### Background

Cognitive impairment represents one of the core features of schizophrenia [1–3], and has been considered of great relevance since the earliest conceptualizations of the disorder [4, 5]. In people living with schizophrenia, several domains appear to show various degrees of deficits, including neurocognitive domains such as attention, speed of processing, verbal and visual memory, working memory, and executive functions [6–8] as well as social cognition domains, such as emotion processing, attributional style, theory of mind and social perception [9–11], and metacognition [12–14]. These impairments are present since an early age, often predating the clinical onset of the disorder [15–18], and can be also observed, albeit in an attenuated form, in nonaffected relatives of people living with schizophrenia [19, 20]. Although a high degree of interindividual heterogeneity can be observed in the severity of the impairments [21], they are more evident in acute phases and appear to be substantially stable over the course of the illness, with the exception of working memory and social cognition, which seem to be more severely affected in chronic stages, suggesting the presence of limited but progressive deterioration [22].

Cognitive deficits have an important negative impact on the lives of people diagnosed with schizophrenia: they greatly interfere with real-world functioning, even more than positive and negative symptoms, producing significant distress also in phases of clinical remission [23–27]. In particular, impairment in neurocognitive performance has negative consequences on functional capacity and on community functioning, on important outcomes such as work success and the ability to live independently, and on determinants of real-world outcomes such as internalized stigma [2, 24–29]. Impairment in social cognition performance has a similar negative impact on community functioning, and perhaps an even greater negative effect on social skills and interpersonal relationships [30, 31]. Moreover, cognitive deficits concur both directly and indirectly in reducing the quality of life of people living with schizophrenia [32–34].

Cognitive impairment also has a negative impact on engagement of the user with mental health services and represents one of the main limiting factors for the process of recovery in the context of psychiatric rehabilitation [35–37].

While accurate and elaborate measuring instruments, as well as rapid and practical screening tools, are available to assess neurocognitive abilities and the majority of social cognition domains [38], pharmacological treatment options appear to be somehow limited, as currently available molecules provide only minimal improvements in cognitive performance. However, several nonpharmacological interventions have been developed, with various amounts of evidence attesting to their effectiveness in providing measurable cognitive gains [3].

In this perspective, the Schizophrenia Section of the European Psychiatric Association (EPA) proposed the development of a guidance paper aimed to provide recommendations for the treatment of cognitive impairment in people living with schizophrenia.

### Aims

The aim of the present work is to present a comprehensive and detailed meta-review of currently available evidence-based treatments for cognitive impairment in people living with schizophrenia and provide recommendations for their implementation both in research settings and in everyday clinical practice.

The guidance will be structured into three sections:

1. Pharmacological treatment, focusing on the effects on cognitive performance of antipsychotics, as well as of other molecules used in the treatment of patients living with schizophrenia.
2. Psychosocial interventions, detailing the effects of cognitive remediation (CR), physical exercise, lifestyle interventions, and other evidence-based psychosocial interventions for the treatment of cognitive impairment in schizophrenia.
3. Somatic treatments, focusing on the effectiveness of cognitive impairment in schizophrenia of noninvasive brain stimulation techniques such as electroconvulsive therapy (ECT), transcranial direct current stimulation (tDCS), and repetitive transcranial magnetic stimulation (rTMS).

## Methodology

### Systematic literature search

The development of EPA guidance on the treatment of cognitive impairment in schizophrenia followed the standardized methods defined by the European Guidance Project of the EPA, as described in previous publications [39–44], and is based on a systematic literature search performed according to the Preferred Reporting Items for Systematic reviews and Meta-Analyses (PRISMA) indications [45, 46].

In line with previous EPA treatment guidance papers [44, 47], a meta-review was conducted to investigate potential treatments for cognitive deficits in schizophrenia. The literature search was conducted on January 11, 2022 on three electronic databases (PubMed, Scopus, and PsycINFO), using the following research string: (Schizophrenia OR “psychosis” OR “psychotic”) AND ((cognit* OR “processing” OR “attention” OR “memory” OR “executive”) AND (“remediation” OR “rehabilitation” OR “enhancement” OR “training” OR “treatment” OR “therapy” OR antipsychotic* OR “molecule” OR “stimulation” OR “technique” OR “intervention” OR “exercise”)) AND (“meta-analysis” OR “systematic review”). No limitation regarding the starting date of the systematic search was applied. A further manual search was conducted on Google Scholar using the key terms of the search string and reference lists of included works were also manually inspected. Studies were selected for inclusion in the EPA guidance according to predefined criteria.

### Selection procedure

To be considered for inclusion, reports had to be meta-analyses or systematic reviews regarding the treatment of cognitive deficits in people living with schizophrenia.

No limitation to inclusion was applied regarding the criteria adopted in individual studies to define the diagnosis of schizophrenia. Records were included also if participants with a diagnosis of schizophrenia did not represent the entirety of the included population, so long as studies conducted on people living with schizophrenia were separately analyzed or discussed.

Documents focusing on psychosocial interventions, physical exercise and lifestyle interventions, noninvasive brain stimulation as well as pharmacological treatment that featured cognitive performance as an outcome of interest were all considered valid for inclusion. Both neurocognition and social cognition were taken into account as outcomes of interest. Reports were considered for inclusion if published in peer-reviewed journals in English language.

Review protocols and nonsystematic reviews were excluded. Systematic reviews including a single study were also excluded.

All documents were independently inspected by at least two screeners and discrepancies in the selection process were discussed and resolved with the support of a third researcher. Data extraction was also independently performed by two researchers.

**Figure 1.**
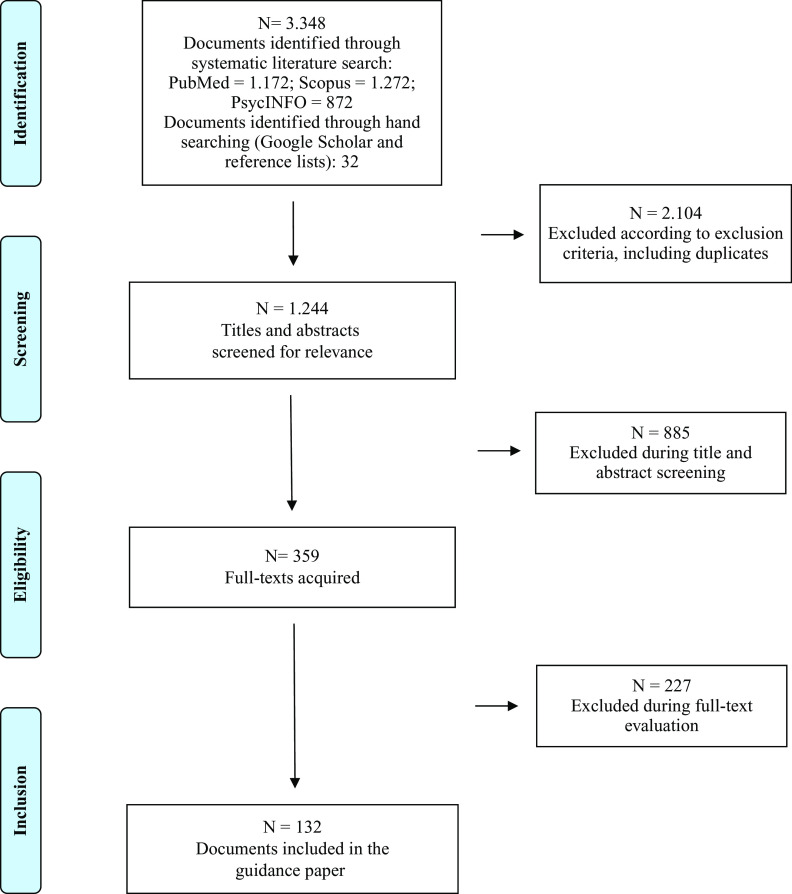
PRISMA flow diagram.

Results of the selection procedure are shown in Figure 1.

### Grading of evidence

Included documents were graded regarding the level of evidence provided, according to previous literature [40]. Grades were assigned according to the indications detailed by Gaebel et al., [39] and modified by Galderisi et al. [43]. Grading criteria for included evidence are reported in Table 1. Discrepancies in the ratings were resolved by discussion among all coauthors.

**Table 1.** Grading of evidence.

Grade	Features of quantitative studies	Features of reviews
I: Generalizable studies	Randomized controlled trials. Surveys sampling a large and representative group of persons from the general population or from a large range of service settings. Analytic procedures are comprehensive and clear usually including multivariate analyses or statistical modeling. Results can be generalized to settings or stakeholder groups other than those reported in the study	Systematic reviews or meta-analyses
II: Conceptual studies	Uncontrolled, blinded clinical trials. Surveys sampling a restricted group of persons or a limited number of service providers or settings. May be limited to one group about which little is known or a number of important subgroups. Analytic procedures are comprehensive and clear. Results have limited generalizability	Unsystematic reviews with a low degree of selection bias employing clearly defined search strategies
III: Descriptive studies	Open, uncontrolled clinical trials. Description of treatment as usual. Survey sampling is not representative since it was selected from a single specialized setting or a small group of persons. Mainly records experiences and uses only a limited range of analytical procedures, like descriptive statistics. Results have limited generalizability	Unsystematic reviews with a high degree of selection bias due to undefined or poorly defined search strategies
IV: Single case study	Case studies. Provides survey data on the views or experiences of a few individuals in a single setting. Can provide insight in unexplored contexts. Results cannot be generalized	Editorials

### Grading of recommendations

Based on the evidence provided by studies analyzed in the included documents, recommendations were developed and reviewed by all coauthors. Grades were then assigned to recommendations according to the indications detailed by Gaebel et al., [39] and modified by Galderisi et al. [43]. Grading criteria of recommendations are reported in Table 2.

**Table 2.** Grading of recommendations.

Grade	Description
A	At least on study or review rated as I and directly applicable to the target population OR a body of evidence consisting principally of studies and/or reviews rated as I, directly applicable to the target population, and demonstrating overall consistency of results
B	A body of evidence including studies and/or reviews rated as II, directly applicable to the target population, and demonstrating overall consistency of results OR extrapolated evidence from studies and/or reviews rated as I or II
C	A body of evidence including studies and/or reviews rated as II–III, directly applicable to the target population, and demonstrating overall consistency of results OR extrapolated evidence from studies and/or reviews rated as II or III
D	Level of evidence rated as III or IV OR extrapolated evidence from studies and/or reviews rated as III or IV OR expert consensus

## Pharmacological Treatment

### Antipsychotic medications

Antipsychotic treatment provides substantial benefits on symptom dimensions in schizophrenia and represents the cornerstone of clinical stabilization, which is in turn a necessary condition to address cognitive impairment and to realize a structured and effective rehabilitation program [48]. In fact, stable antipsychotic treatment is required to avoid symptom exacerbations and avoid relapses [49], and, if stabilization is maintained for a sufficiently long period of time, allows the implementation of nonpharmacological interventions, with important repercussions not only on the patients’ real-world outcomes, but also on their quality of life [50], global health and even mortality [51, 52].

To date, 15 systematic reviews and meta-analyses have been published regarding the effects on cognition of antipsychotic treatment (Supplementary Table 1).

A recent and comprehensive assessment of the effects of antipsychotic treatment on cognitive performance [53] included 42 randomized double-blind controlled trials with three or more weeks of follow-up, for a total of 5,866 participants. Both head-to-head comparisons and placebo-controlled studies were included; 10 network meta-analyses were performed, with no inconsistencies emerging between direct and indirect comparisons in all networks. Favorable effects were observed for amisulpride, quetiapine, lurasidone, olanzapine, perphenazine, risperidone, sertindole, and ziprasidone, with small differences between molecules emerging in the different cognitive domains. Inferior effects were observed for remoxipride, clozapine, and haloperidol, outperformed by placebo in most cognitive domains, as well as in the composite score evaluating global cognitive effects. In light of these results, clozapine treatment should be evaluated with great caution, considering also that this molecule is approved only for treatment-resistant patients, in which, however, it may provide significant clinical benefits. The positive effect of perphenazine is discussed as an unexpected result by the Authors of the meta-analysis, and could be largely dependent on the influence of the CATIE study data [54].

A recent meta-analysis including 19 studies and comparing the effects on cognition of different categories of second-generation antipsychotics [55] reported that both chemical categories (-pines and -dones) produced small significant positive effects on attention, working memory, executive functions, motor function, nonverbal memory, processing speed, and verbal memory, and no significant differences between categories were observed.

Another meta-analysis [56] focused on cognitive effects of second-generation antipsychotics compared to placebo: nine trials for a total of 1,111 participants were analyzed and a small significant pro-cognitive effect was observed for second-generation antipsychotics.

Previous meta-analyses reported similar results, with second-generation antipsychotics emerging as consistently superior to first-generation ones, and showing small improvements in global cognitive performance and single cognitive domains, including social cognition, with no molecule consistently outperforming the others [57–62].

A recent and well-conducted meta-analysis [63] compared oral and long-acting injectable formulations of antipsychotics on a wide range of outcomes (efficacy, effectiveness, hospitalizations, adverse events, cognition, functioning, and quality of life) and included 137 studies (randomized controlled trials, cohort studies, and pre–post studies) totaling 397.319 patients. While long-acting formulations were found to be superior to oral formulations in terms of risk of hospitalizations and relapse, no significant differences were observed regarding cognitive performance: out of 19 included studies considering this outcome, 2 reported a superior effect of long-acting formulations, 1 a superior effect of oral formulations and 16 reported no differences.

Finally, newer second-generation antipsychotic molecules such as cariprazine, brexpiprazole, and lumateperone have shown some promising but preliminary findings of efficacy on cognitive performance; however, further studies are required to better assess the entity of such improvements [64–67].

### Recommendations

Considering the available literature, the working group elaborated the following recommendations:

**Table:** 

Grade	Recommendation 1a
A	Second-generation antipsychotics are recommended for their favorable cognitive profile compared to first-generation antipsychotics

**Table:** 

Grade	Recommendation 1b
A	For patients with cognitive impairment who are treated with a first-generation antipsychotic, a switch to a second-generation antipsychotic should be considered

**Table:** 

Grade	Recommendation 1c
B	No clear superiority of a single second-generation antipsychotic over other molecules of the same category has currently been found regarding cognitive outcomes

### Other pharmacological treatments

Systematic reviews and meta-analyses on the effectiveness of other pharmacological therapies to address cognitive deficits in schizophrenia are reported in Supplementary Table 2.

Regarding other pharmacological agents that are routinely used in clinical practice in the treatment of schizophrenia spectrum disorders, anticholinergic medications have been shown to be correlated with negative cognitive outcomes in several recent large and well-conducted trials, with anticholinergic burden increasing cognitive impairment [68–72] and limiting the positive effect of therapeutic interventions on cognition [73–75]. The results of a recent study also suggested that anticholinergic burden can have a direct negative impact on functional capacity [76]. However, while systematic assessments of available literature confirm the negative effect of anticholinergics in older adults and in poly-pathological patients [77–79], no meta-analyses or systematic reviews regarding the effect of anticholinergic medications or anticholinergic burden in people living with schizophrenia are available.

Therefore, more studies, including systematic assessments of available literature, are currently needed, but, considering currently available evidence, it is advisable to keep anticholinergic burden to a minimum, particularly in long-term treatment and in patients showing prominent cognitive impairment.

Benzodiazepines are also often used in the treatment of schizophrenia and could have a negative impact on cognition: recent studies have shown a negative impact of benzodiazepines on some domains of cognition [80], and could represent a specific factor contributing to vulnerability to cognitive deterioration in older adult patients [81]. However, no systematic assessment of their impact on cognition in people living with schizophrenia is currently available.

Regarding antidepressants, a recent meta-analysis [82] considering the effects of adjunctive fluvoxamine, included five randomized controlled trials (284 participants), two of which also evaluated cognitive outcomes, and observed no significant pro-cognitive effect of the antidepressant drug. A Cochrane Collaboration meta-analysis [83] focused on adjunctive mirtazapine and included nine randomized controlled trials (310 participants): four studies reported data on cognitive outcomes, three of which were included in the systematic evaluation of cognition, and, although minimal positive effects were reported in one trial, no substantial improvement in cognition was observed. These results are in line with those of a previous meta-analysis [84] exploring the pro-cognitive effects of mirtazapine, citalopram, fluvoxamine, duloxetine, mianserin, bupropion, and reboxetine, including 11 studies and 568 participants: a statistically significant positive effect was observed for pooled antidepressants compared to placebo on both global cognition and executive functions, but the dimension of the effect was too small to be regarded as clinically meaningful. Similar results were observed in a meta-analysis [85] considering different types of pharmacological augmentation to antipsychotic treatment: a significant positive effect was observed for pooled antidepressants, but the size of the positive effect was minimal.

Several other pharmacological agents are currently being evaluated for the treatment of cognitive deficits in people with schizophrenia. Different molecules are in various phases of preclinical and clinical evaluation, while systematic assessment of current evidence is already available for others (for a complete list, see Supplementary Table 2). Among these, the most promising seems to be *N*-acetylcysteine [86, 87], which appears to have neuroprotective effects and regulate glutamatergic pathways by acting on the redox/glutathione sensitive site of the *N*-methyl-d-aspartate receptors; D-serine [88], also acting on the glutamatergic pathway; memantine [89, 90], as well as other molecules targeting the *N*-methyl-d-aspartate receptors [85].

Among molecules targeting the cholinergic system, acetylcholinesterase inhibitors [91, 92] have been evaluated and found not to provide improvements in cognitive performance of patients with schizophrenia, but small positive effects have been observed with galantamine [93]. Alpha-7 nicotinic receptors agonists [94] and positive allosteric modulators [95] do not appear to provide substantial benefits.

Cannabidiol represents another molecule that has been the object of some studies but currently does not appear to have a significant clinical effectiveness [96, 97].

Anti-inflammatory drugs and immunomodulators represent other categories of molecules that could act as positive modulators of cognitive performance in people living with schizophrenia and are currently object of investigation [98, 99].

Intranasal oxytocin as well as other molecules are currently being assessed [100, 101] as potential treatments for social cognitive deficits.

However, all these molecules are not currently approved for the treatment of schizophrenia spectrum disorders and should be considered as off-label treatments.

## Psychosocial Interventions

### Cognitive remediation

The psychosocial intervention targeting cognitive impairment in schizophrenia with the largest amount of currently available evidence of efficacy is CR.

CR, according to the latest definition proposed by the CR Experts Workshop, is a behavioral training–based intervention that aims to improve cognitive processes with the goal of durability and generalization [102]. Based on these principles, several different interventions that fit the definition of CR have been developed and can be subdivided in bottom-up or top-down, individual- or group-based, pencil-and-paper or computerized, or include different combinations of these elements. Structure and frequency of sessions and duration of treatment programs can also vary across the different interventions [103].

To date, 25 systematic reviews and meta-analyses focused on CR and related interventions have been published (Supplementary Table 3).

The most recent and comprehensive meta-analysis on the effectiveness of CR for people living with schizophrenia included 130 studies and a total of 8,851 participants, and found a consistent small-to-moderate positive effect of CR on cognitive performance and functioning [104].

These results are in line with the findings observed in the earliest meta-analyses on CR effectiveness [105–107] and are confirmed by other very recent and methodologically rigorous meta-analyses, reporting very similar results also when separately considering interventions targeting cognition (73 studies, 4,594 participants) [108] and social cognition (42 studies, 1,868 participants) [109]. These results are also in line with those reported in other recent meta-analyses and systematic reviews focusing on social cognitive training [110, 111].

Of interest, a multi-outcome meta-analysis including 67 studies on computerized CR and 4,067 participants highlighted that functional improvement depends on cognitive gains, confirming the theoretical principle that restoring cognitive abilities would result in better functional outcomes [112].

Another recent meta-analysis, updating the search of the most comprehensive systematic assessment of CR effects, investigated the acceptability of CR interventions expressed as treatment drop-out rates [113]: CR emerged as a treatment that is well-accepted by participants, with attrition rates that are similar to those of other psychosocial interventions.

Given this impressive wealth of evidence and considering the coherence of results of multiple independently conducted large meta-analytic studies, the efficacy and the effectiveness of CR can be considered by the research community as undeniable [114].

However, ingredients of effectiveness, moderators of response, as well as barriers and facilitators for implementation in real-world clinical practice of rehabilitation services represent fundamental issues that require further discussion and investigation. In the largest meta-analysis, no difference in effectiveness was observed regarding pencil-and-paper or computer-delivered interventions, or regarding individual- or group-based programs. On the contrary, the active participation of a trained therapist, the repetition of cognitive exercises, the development of novel cognitive strategies, and the presence of activities to facilitate transfers of cognitive gains in the real-world context emerged as core elements of effectiveness. In particular, the integration of CR into a structured psychiatric rehabilitation program or its association with other evidence-based psychosocial interventions produced better improvements in both cognition and functioning [104]. In fact, a recent meta-analysis including 23 studies with 1.819 participants was focused on the combination of CR with psychiatric rehabilitation and found a significant synergic effect on vocational and social functioning [115], while another meta-analysis reported better cognitive gains in interventions that utilize bridging groups and strategy-coaching [108]. Moreover, factors enhancing efficacy also increase influence treatment acceptability [113].

Taken together, these findings show that, while the different CR programs can be considered as equally effective, CR should be offered to people living with schizophrenia not only as a stand-alone and isolated treatment; instead, it should be provided in mental health services, where sufficient resources are available, in the framework of a structured rehabilitation project and delivered by a trained therapist with the aim of developing novel cognitive strategies and applying them in the real-world.

As regards optimal candidates for CR interventions, the largest meta-analysis found that participants with fewer years of education and higher baseline symptoms severity showed larger improvements in cognitive performance, while fewer years of education and lower premorbid IQ emerged as positive moderators of functional improvement [104]. Similarly, fewer years of education were associated with greater social cognitive gains in CR interventions targeting social cognition [109]. Moreover, a recent meta-analysis including 20 studies for a total of 1,509 participants, found that CR has a consistent positive effect on cognition also in inpatients, which usually show a more severe clinical condition [116], while another meta-analysis of 11 studies with 615 participants found a significant positive effect of CR in patients with early schizophrenia but a smaller magnitude of cognitive improvement if compared to that observed in more chronic patients [117].

Taken together, these findings suggest that more substantial gains can be observed in more clinically compromised participants, probably as they present larger room for improvement. However, a recent systematic review focusing on moderators of response, including 40 studies and 1,681 participants, highlighted a large degree of inconsistency in the results of single studies, with the vast majority reporting no association between investigated variables and response to treatment [118]. Therefore, individual characteristics of participants do not appear to act as a barrier to obtain significant benefits from CR. In this perspective, CR represents an intervention that has the potential to be proposed to all users of mental health services with a diagnosis of schizophrenia.

While recent evidence suggests that CR interventions can also be delivered remotely, attrition rates appear to be very high, and more research is currently needed to confirm its effectiveness in this format: in-person treatment sessions currently represent the optimal standard [119].

### Recommendations

Considering the available literature, the working group elaborated the following recommendations:

**Table:** 

Grade	Recommendation 2a
A	Cognitive remediation is recommended for the treatment of cognitive impairment in people living with schizophrenia

**Table:** 

Grade	Recommendation 2b
A	Social cognitive training is recommended for the treatment of social cognitive deficits in people living with schizophrenia

**Table:** 

Grade	Recommendation 2c
A	Cognitive remediation interventions should be delivered by a trained therapist and integrated in a psychosocial rehabilitation program

### Physical exercise and lifestyle interventions

Physical exercise can be considered an intervention providing substantial benefits for people living with mental disorders [120]. In fact, physical activity is recommended as an evidence-based treatment for severe mental illnesses according to a published EPA guidance [47]. It is recommended as a treatment for mild–moderate depression to improve symptoms and physical fitness with the highest recommendation grade. Regarding schizophrenia spectrum disorders, physical exercise is currently recommended as an adjunctive treatment to improve symptoms, cognitive performance, and quality of life, albeit with a lower level of recommendation.

To date, 11 systematic reviews and meta-analyses including data on various kinds of physical exercise and lifestyle interventions in people living with schizophrenia and considering cognition as a treatment outcome have been published (Supplementary Table 4).

The most pertinent, comprehensive, and recent meta-analysis [121] on the effects of physical exercise on cognitive performance in people with schizophrenia included 10 controlled trials and a total of 385 participants. A significant small-to-moderate positive effect was observed on global cognitive performance, with no significant statistical heterogeneity; a significant positive effect with a moderate effect size was observed in the sensitivity analysis including only randomized controlled trials (seven studies, 297 participants). Considering separate cognitive domains, significant positive effects were observed in working memory, albeit with considerable statistical heterogeneity (seven studies, 282 participants), attention/vigilance (three studies, 104 participants), and social cognition (three studies, 81 participants), while no effect was observed in processing speed (six studies, 195 participants), verbal memory (six studies, 166 participants), visual memory (three studies, 61 participants), and reasoning and problem solving (four studies, 146 participants) domains. No significant moderator of effectiveness emerged in the dedicated analyses, but greater amounts of exercise in minutes of activity per week of treatment were correlated with larger cognitive gains with trend-level significance (*p* = 0.065). Also, a larger effect was observed in studies where physical activity was supervised by a trained professional, but the subgroup analysis did not reach statistical significance. Interestingly, three included studies for a total of 76 participants compared a combination of physical exercise and CR to CR alone, and in the quantitative synthesis, the hypothesized superiority of the combined treatment did not reach statistical significance. This result, however, could also be determined by the low number of participants included in the analysis.

All these findings are in contrast with the results of a previous meta-analysis [122] that reported improvements in clinical symptoms, quality of life, global functioning, and depressive symptoms, but failed to observe a significant effect of physical exercise on cognitive performance. However, only separate cognitive domains were analyzed in this work, and only six studies were included reporting data on cognitive outcomes. Another previous meta-analysis included only a single study reporting effects on cognitive outcomes [123].

A systematic review investigated the biological mechanisms that could be involved in the positive cognitive effects observed as a result of physical exercise [124]. Fourteen trials for a total of 423 participants were included in the review, seven reporting neuroimaging data and seven focusing on peripheral biomarkers. Neuroimaging studies mostly reported changes in total gray matter volume and volume changes in the hippocampal region that were correlated with cognitive improvement. One functional Magnetic Resonance Imaging study reported increased activation in the extrastriate body area of posterior temporal cortex following sport-related visual stimuli in the exercise group in a 3-month follow-up. Biomarkers studies mostly showed that an increase in the peripheral levels of Brian-Derived Neurotrophic Factor correlated with cognitive gains; two studies investigated inflammatory markers (CRP, IL-6, and TNF-α), but did not observe significant differences between treatment and control groups. No change was also observed in IGF-1, while one study found a small increase in salivary cortisol levels and working memory performance in the intervention group.

A recent meta-analysis [125] included 59 randomized controlled trials of interventions based on exercise or psychotherapy focused on changes in diet and physical activity, including also yoga and tai-chi, with participants diagnosed with schizophrenia spectrum disorders. While 10 different included studies provided results on cognitive outcomes, only 2 had their results pooled together, as they both used forward and backward digit span test of the Wechsler Adult Intelligence Scale, and reported a significant small positive effect of moderate-vigorous aerobic exercise and mind–body exercise that was not maintained at follow-up evaluations; the other studies, each using different tests and targeting different cognitive domains, were not included in the quantitative synthesis, but six of them observed significant positive effects in the explored domains.

Another recent meta-analysis [126] included 122 studies on physical exercise as an add-on therapeutic intervention in participants with Alzheimer’s disease, Huntington’s disease, multiple sclerosis, Parkinson’s disease, schizophrenia, and unipolar depression; quantitative synthesis was subdivided on the basis of outcomes and diagnostic categories, with eight studies including participants with schizophrenia spectrum disorders. A significant moderate positive effect was observed in the psychomotor speed domain but only two studies with a total of 120 participants were included in this analysis. On the other hand, no significant effect was observed in attention and working memory (four studies, 557 participants), executive functioning (two studies, 388 participants), and memory (three studies, 406 participants), and no analysis was conducted on global cognition in people living with schizophrenia.

Interestingly, a meta-analysis compared mindful exercise (yoga, tai-chi, and qicong) and physical exercise. It included seven studies with 679 total participants [127] and analyzed cognitive performance as an outcome of interest; however, only three studies provided relevant data and no significant superiority of mindful exercise in cognitive gains emerged from the observed results, with only one study showing grater working memory improvement in yoga compared to aerobic physical exercise.

A recent systematic review focused on dance movement therapy for people with different psychiatric disorders, including 15 studies and 860 participants [128]: five studies included participants diagnosed with schizophrenia, and two studies reported positive effects on cognitive outcomes.

Regarding lifestyle interventions beyond physical activity, a recent systematic review [129] investigated the impact of diet modifications on clinical symptoms, cognitive performance, and quality of life in people diagnosed with schizophrenia spectrum disorders, including 25 trials and 4,448 participants. A high degree of heterogeneity in trial designs, interventions, recruited samples, and treatment outcomes was observed. Only three studies for a total of 446 participants investigated cognitive outcomes in adult patients diagnosed with schizophrenia spectrum disorders: a small study (10 participants) that involved group nutrition education, CR, social skills, and meal preparation and reported cognitive improvement, a small study (eight participants) on a group educational program including nutritional balance, meal planning, budgeting, meal preparation and socialization, and one large study (428 participants) on an individual lifestyle program including a healthy diet, cooking, smoking cessation, physical activity and coordination of care for somatic health reporting no significant cognitive gains.

In conclusion, while previous reports highlighted more conflicting results from primary studies, recent findings suggest that physical exercise interventions can be considered an evidence-based treatment to improve cognition in people living with schizophrenia. More research is currently needed to establish which modalities, intensity, and duration of interventions produce greater benefits, but the active participation of a trained instructor appears to represent an ingredient of efficacy. As 150 min of moderate to vigorous physical activity per week is recommended to be integrated in multidisciplinary treatment programs to provide substantial benefits in multiple clinically relevant domains in people living with schizophrenia [47], this can be considered the standard amount of exercise than can be recommended in rehabilitation practice.

While there is a clear rationale for a positive effect of diet and other lifestyle intervention in the integrated treatment of people living with schizophrenia, considering also that cardiovascular risk factors such as metabolic syndrome, hypertension, and diabetes are strongly correlated with cognitive impairment in this population [130], more research is currently needed to properly assess the impact of these interventions on the treatment of cognitive deficits. In this regard, future research should aim to provide reliable results that can produce meaningful qualitative and quantitative syntheses: to this end, current recommendations on the assessment of cognitive abilities in people living with schizophrenia could represent a valid support [38].

### Recommendations

Considering the available literature, the working group elaborated the following recommendations:

**Table:** 

Grade	Recommendation 3a
B	Physical exercise should be integrated into rehabilitation projects considering its positive effects on cognition

**Table:** 

Grade	Recommendation 3b
C	Lifestyle interventions could have mild positive effects on cognitive functioning

### Other psychosocial interventions

Several psychosocial interventions have shown consistent positive effects in clinically relevant areas when implemented in the treatment and in the rehabilitation process of individuals diagnosed with schizophrenia.

Social skills training is effective in reducing negative symptoms, general psychopathology severity, and total symptoms severity in people with schizophrenia, and provides also substantial improvements in social performance [131, 132].

Cognitive behavioral therapy for psychosis has been shown to improve real-world functional outcomes, including work and social functioning [133–136].

Psychoeducation is effective in reducing relapses and ameliorates caregiver’s burden and measures related to overall wellbeing [137, 138].

However, the impact of these treatments on cognitive performance is seldom evaluated, and systematic assessment of effectiveness on cognitive impairment is available only for a limited number of interventions (Supplementary Table 5).

Compensatory interventions for cognitive impairment in psychosis have been confirmed to be effective in producing small-to moderate functional gains in a recent meta-analysis of 25 randomized controlled trials and a total of 1,654 participants [139]; however, cognitive performance was not considered among the outcomes of the study.

A recent systematic review on immersive virtual reality in people living with schizophrenia included six studies [140] and reported positive results from two trials focused on treatment of cognitive deficits. A previous well-conducted systematic review on the assessment and treatment of psychosis with virtual reality included 50 studies [141] and reported that this method can be useful not only in assessing the entity of cognitive impairment, but also in delivering treatment, including evidence-based interventions. A previous Cochrane Collaboration review [142] reported negative findings but only three studies were included in this investigation.

A recent Cochrane Collaboration review [143], including seven trials and 468 participants focused on video games for people with schizophrenia, usually included as controls in trials evaluating the effects of computerized interventions: unsurprisingly, they showed no significant effect of video games on cognitive outcomes. Actually, video games were inferior as compared to evidence-based treatments, such as CR.

A systematic review focusing on mindfulness-based interventions for people living with severe mental illnesses [144] included seven studies, all involving participants diagnosed with psychotic disorders. Among these, only one study, including 10 participants, considered cognitive performance as a treatment outcome, measured with the MATRICS Consensus Cognitive Battery, and reported a significant positive effect in the working memory domain.

A Cochrane Collaboration review [145], updating a previous work [146], explored the effects of music therapy in the treatment of schizophrenia and included 18 studies and 1,215 participants. Only three studies assessed cognitive functioning, reporting conflicting results in the attention/vigilance domain and positive effects in short-term working memory and on long-term abstract thinking capacity.

Overall, the working group considered the psychosocial interventions reported above as potential promising approaches to the treatment of cognitive impairment in schizophrenia but did not consider the available evidence sufficient for a recommendation.

## Somatic Interventions

### Noninvasive brain stimulation techniques

Brain stimulation techniques are based on the principle of modulating brain activity through the use of magnetic or electric induction. Invasive brain stimulation techniques are defined as such as they require an elective surgical procedure to be delivered and include deep brain stimulation and vagus nerve stimulation. Noninvasive brain stimulation treatments, on the contrary, include treatments that can be delivered without the risks and the adverse effects of invasive surgery and include ECT, TMS, and tDCS [147].

ECT entails the induction of generalized cerebral seizures under general anesthesia, producing brain chemistry changes by influencing neurogenesis, neurotrophic signaling, and neuroplasticity [148]. ECT, however, is frequently associated with cognitive adverse effects, including transient cognitive impairment and memory deficits [149, 150]. TMS is based on the electromagnetic induction principle: focal electromagnetic pulses penetrate the skull through a wire coil to focally stimulate target areas by inducing secondary electric current flows modulating neuronal firing rates [151]. tDCS consists in applying low-amplitude direct currents (usually 1–2 mA) through anode and cathode electrodes applied to the scalp, modulating cortical excitability in a more nonfocal way by polarity-dependent shifts of neuronal membrane potentials [152].

Recent meta-analytic findings have shown promising results regarding the effectiveness of noninvasive brain stimulation, particularly on TMS and tDCS, on treating core symptom dimensions of schizophrenia [153]. As to the effectiveness of different noninvasive brain stimulation techniques on cognition, to date 22 meta-analyses and systematic reviews have been published (Supplementary Table 6).

Regarding ECT, a recent systematic review [154] included 24 different studies on ECT in people living with schizophrenia, with five studies reporting data on cognitive outcomes. Conflicting results regarding cognitive adverse effects are reported, with three studies finding no significant long-term worsening of cognitive symptoms and one study even reporting a positive effect on this outcome.

Another systematic review [155] investigated whether stimulus parameters and electrode placements could have a role in determining cognitive side effects and included three randomized, double-blind, clinical trials, one randomized, nonblinded trial, and one retrospective study. This review, again, reported conflicting results, with very limited findings suggesting more favorable outcomes of right unilateral placing of electrode compared to bilateral. A recent Cochrane Collaboration Review [156] focused on ECT in treatment-resistant schizophrenia, including 15 studies involving 1,285 participants: no study reported a clinically relevant change in participants’ cognitive functioning, while one study reported the incidence of short‐term memory deterioration. Similar results are reported in a previous meta-analysis [157].

Regarding the effects of tDCS and TMS, several systematic reviews and meta-analyses have been recently published. Among the most pertinent, comprehensive, and recent assessments, a meta-analysis [158] included 82 studies for a total of 2,784 participants and investigated the effects of both tDCS and TMS in improving cognition in schizophrenia, depression, dementia, Parkinson’s disease, stroke, traumatic brain injury, and multiple sclerosis. A total of 24 studies recruited participants diagnosed with schizophrenia, 14 (672 participants) for TMS and 10 (314 participants) for tDCS. Pooling together the results observed in the different clinical conditions, a minimal to small positive effect was observed for both TMS and tDCS on the working memory domain and for tDCS on the attention/vigilance domain. No significant effect was observed in all other cognitive domains and no significant improvement was detected when only studies with schizophrenia-diagnosed participants were included. Another meta-analysis [159] explored the effects of TMS and transcranial electric stimulation (tES, including both tDCS and transcranial alternate current stimulation or tACS) on the working memory of people living with schizophrenia: 22 studies were included in the review, 9 (381 participants) for TMS and 13 (327 participants) for tES (12 studies for tDCS, 1 study tDCS, and tACS). A high degree of heterogeneity in techniques and outcomes measurements was observed, and no significant positive effect was found for any of the interventions. These results are in line with those of a systematic review focused on transdiagnostic cognitive effects of repetitive TMS (rTMS) [160] that reported no significant positive effect on cognition in people living with schizophrenia. Another systematic review [161] explored the transdiagnostic effects of rTMS on attention, including four studies (129 participants) in people with schizophrenia: only one study reported a significant positive effect. These findings are in line with previous reports highlighting the lack of significant pro-cognitive effects of TMS [162–164]. Another meta-analysis including nine studies and 351 participants [165], instead, reported that high-frequency rTMS appears to produce a lasting small positive effect on working memory, on the basis of an analysis conducted on seven studies.

Regarding tDCS, a meta-analysis on adjunctive multi-session tDCS in people living with schizophrenia [166] which included 12 randomized controlled trials (418 participants) found a significant small positive effect on working memory, but no effect on other cognitive domains. These results are in line with the results of a previous meta-analysis [167]. Similarly, a meta-analysis focusing on the effects of tES specifically on working memory [168], including 12 studies (429 participants), reported a lasting small positive effect in this specific domain. Another meta-analysis included 14 studies, with seven reporting cognitive outcomes: while a trend for a positive effect on cognition was observed, it failed to reach statistical significance [169]. A recent systematic review [170] focusing on cognitive effects of tDCS for people living with schizophrenia included both randomized controlled trials and other types of studies, for a total of 32 records. The majority of the studies, 21 reports, provided evidence of positive effects of tDCS on various cognitive domains, while 11 provided negative findings. In particular, 12 out of 18 studies reported positive effects on memory, 8 out of 13 on attention and cognitive control, and 2 out of 3 on social cognition. However, no specific tDCS parameters such as electrode montage, stimulation protocol, type, and intensity were clearly associated with positive effects on cognitive impairment. Similar results are discussed in a systematic review [171] that examined the cognitive effects of tDCS across various brain disorders.

Based on the available evidence, even if cognitive impairment does not appear to substantially worsen after ECT, due to the risk of adverse cognitive effects, ECT is recommended to be avoided in people living with schizophrenia with the primary intention of treating cognitive deficits. It can be considered a valid treatment choice in cases of severe catatonia and in subjects that show long-standing resistance to pharmacological treatment: in these cases, particular attention should be dedicated to changes in cognitive performance, and a multidisciplinary approach should be adopted to avoid cognitive deterioration and improve cognitive abilities with the help of evidence-based interventions.

Currently, the available literature does not allow to recommend TMS as an evidence-based treatment for cognitive impairment. Some encouraging findings suggest that tDCS could provide some positive cognitive effects, particularly in the domain of working memory, but it cannot be currently recommended as an evidence-based treatment for cognitive impairment in people living with schizophrenia. In particular, it is not recommended as a stand-alone treatment to be used in clinical practice. More research is currently needed to better assess its effectiveness when integrated in multidisciplinary treatment programs, its optimal treatment parameters and modalities of delivery, and also its impact on functional outcomes.

## Discussion

Considering the available literature regarding the treatment of cognitive impairment in people living with schizophrenia, several advances and significant developments can be observed in some areas, with systematic assessment providing robust evidence of the effectiveness of specific treatments.

Appropriate pharmacological management represents a fundamental starting point in the treatment of schizophrenia, and this holds true also when considering cognitive symptoms. In fact, despite currently available second-generation antipsychotics show only minimal positive effects on cognitive performance in schizophrenia, they present a substantially superior cognitive profile than first-generation ones [53] and may indeed exert a neuroprotective effect that the older molecules do not provide [172]. Nevertheless, a careful management of pharmacologic treatment in the perspective of preserving and improving cognitive functioning should not be limited to the preferred use of second-generation antipsychotics: attention should also be devoted to limit anticholinergic and benzodiazepines burden, particularly in long-term treatment, as they could have a consistent negative impact on cognitive outcomes and even on patients’ functional capacity [71, 76, 80].

Regarding available treatments that have been shown to reliably provide substantial effects in addressing cognitive impairment, psychosocial interventions currently represent the most effective instruments. In fact, CR and related interventions are supported by a recent and robust body of evidence attesting to their effectiveness in improving cognitive outcomes. Their implementation in rehabilitation services and in day-to-day clinical practice is therefore strongly recommended to treat cognitive impairment in people living with schizophrenia and, consequently, improve real-world functioning and achieve important personal goals. Physical exercise-based interventions should also be recommended. Other treatments, including adjunctive pharmacological treatments, novel molecules that are currently in clinical and preclinical evaluation, somatic treatments, as well as other psychosocial interventions that have shown to consistently provide significant benefits in other clinically relevant areas, such as social skills training, cognitive behavioral therapy, and psychoeducation, could be effective in producing cognitive gains, but more research is needed to properly assess their effectiveness on cognitive functioning. It is recommended to always assess cognitive outcomes when considering pharmacological, psychosocial, and somatic treatments for people living with schizophrenia, and to conduct these assessment using methods that allow good reproducibility and synthesis of results [38]. The different approaches devoted to the improvement of cognitive impairment should also be used as soon as possible in the course of the disorder, as this could have a positive longitudinal effect on the trajectory and the outcomes of the illness [173, 174].

As regards the limitations of the present guidance paper, it should be noted that, as a meta-review, result of recent individual clinical studies might have not been taken into account. However, even large and well-conducted studies provide only limited information in the context of developing treatment recommendations and guidance.

The restriction to works published in English language could represent another limitation; however, the influence of this element on the accuracy of systematic literature searches is often described as small and negligible [175, 176].

One important issue that has to be considered regarding the treatment of cognitive impairment in schizophrenia is that even the most well-recognized treatments provide only a small to moderate gain in cognitive performance, and their mechanisms of action are, for the large part, unknown and hypothetical. While it is true that even small improvements in cognition could produce substantial functional gains, this fact should be taken into account, highlighting the need to further develop novel and effective treatments and solutions.

Clinicians and mental health services organizers should pay particular attention to the difficulties in the implementation in real-world settings of psychosocial interventions for cognitive impairment, as they represent one of the major issues when trying to translate the results of randomized controlled trials into real-world clinical practice. To reduce the science-to-service gap, leadership should provide direction, resources, and support, commitment to continuing education of the work-place staff regarding new techniques, and encouragement to learn from direct experimentation [177].

Additional research is currently needed also to establish the optimal treatment intensity, duration, and modalities of delivery of effective treatments: while available literature already provides substantial insight, suggesting that both CR and interventions based on physical exercise yield greater gains when actively delivered by trained professionals and when integrated into structured rehabilitation projects [47, 104, 121], further information is still required to fully optimize these treatments and, more importantly, to tailor personalized rehabilitation programs to each single person with schizophrenia [3]. Personalization still depends on a clear case formulation where individual goals are set to provide an appropriate treatment program [103]. More individualized diagnostic characterization for schizophrenia or other primary psychotic disorders could now be possible with ICD-11 [178, 179], which includes severity-graded, operationalized cognitive symptom specifiers that can also contribute to better-matched treatment selection.

Personalized treatment programs should also carefully take into account the context of participants: family, parents, and siblings could represent important resources also in the perspective of developing treatment programs addressing cognitive impairment. The systematic literature search yielded little evidence regarding this topic, and therefore it deserves more scientific attention.

Another area of research that needs further growth regards the implementation in clinical practice of effective available interventions: a better understanding of which barriers and limitations should be overcome and which facilitators should be in place to promote the implementation of evidence-based interventions might contribute to their translation to the real-world setting of mental health services, with the ultimate goal of providing substantial benefits for people living with schizophrenia.

Finally, future studies should address the issue of the costs of the treatments targeting cognitive impairment, considering a careful calculation of costs and benefits which should also take into account the indirect costs of cognitive deficits and the benefits of treatments in term of relapse prevention and reintegration into working life. The available evidence base for the benefits of interventions targeting cognitive performance in terms of costs is growing [180, 181] and this factor should be more constantly a part of new trial outcomes.

## Conclusions

Cognitive impairment remains a complex issue in people living with schizophrenia, with a substantial negative impact on functional and recovery outcomes. Available evidence-based treatments are currently limited and provide moderate improvements in cognitive performance. Therefore, mental health professionals should provide interventions that limit or eliminate factors that further hinder cognitive functioning in schizophrenia and should widely apply effective available interventions. More research on treatment of cognitive impairment in schizophrenia is anyway needed, both aimed at developing novel effective treatments, and at implementing, optimizing, and personalizing those already available.

## Data Availability

All the data that support the findings of this study are available within the article and its supplementary material.

## Supplementary material

For supplementary material accompanying this paper visit https://doi.org/10.1192/j.eurpsy.2022.2315. click here to view supplementary material
