# Supplementary material for: European Psychiatric Association guidance on treatment of cognitive impairment in schizophrenia
Source: Eur Psychiatry. 2022 Sep 5;65(1):e57. doi: 10.1192/j.eurpsy.2022.2315 (PMC9532218; doi:10.1192/j.eurpsy.2022.2315)
Supplement: Supplementary file 1 [file S092493382202315Xsup001.docx]

**Index:**

**eTable 1. Pharmacological treatments – Antipsychotic Medications**

**eTable 2. Pharmacological treatments – Other Pharmacological Treatments**

**eTable 3. Psychosocial interventions - Cognitive Remediation**

**eTable 4. Psychosocial interventions - Physical Exercise and Lifestyle Interventions**

**eTable 5. Psychosocial interventions - Other Psychosocial Interventions**

**eTable 6. Somatic interventions- Non-invasive Brain Stimulation Techniques**

**References**

**eTable 1. Pharmacological treatments – Antipsychotic Medications**

| **Authors** | **Type of Document** | **Types of Included Studies** | **Level of evidence** | **Number of Included Studies**  **(Number of participants and diagnoses)** | **Intervention** | **Main Findings** |
| --- | --- | --- | --- | --- | --- | --- |
| Keefe RS et al., 1999. [1] | Meta-Analysis | RCTs and open label studies | I | 15 (SCZ) | Atypical antipsychotics | The meta-analysis of the three double-blind studies indicated that atypical antipsychotics were significantly more effective than conventional antipsychotics at improving cognitive functioning (X2 = 14.82, p = 0.022). Meta-analysis of the 12 open-label studies supported the results of the double-blind studies. In these studies, atypical antipsychotics also improved neurocognitive functions (X2 = 47.59, p = 0.002). Meta-analytic procedures that included all 15 studies also supported the effect of atypical antipsychotics on cognition (X2 = 62.41, p = 0.0004). |
| Kennedy E et al., 2000. [2] | Systematic Review | RCTs | I | 23  (4445 SCZ) | Risperidone | Little can be concluded about the long-term effects of risperidone and generalizing results beyond a comparison with haloperidol would be imprudent. For outcomes of poor concentration, risperidone is not different to haloperidol (n=1548, 4 RCTs, RR 0.90, 95% CI = 0.8 to 1.1), and the outcome for poor memory is similar (n=1469, 2 RCTs, RR 0.84, 95% CI = 0.7 to 1.0). |
| Mishara AL et al., 2004. [3] | Meta-Analysis | Controlled trials | I | 34  (1026 SCZ) | FGAs | Typical antipsychotic medications provide modest-to-moderate gains in multiple cognitive domains. ES were generally in the 0.13-0.29 range for the majority of the cognitive functions. One effect size, automaticity of learning, had a large ES (<0.80 or greater); one effect size, perceptual processing, had moderate range ES (<0.50), and three other ES (attention, language, and memory) were at the low end of the moderate range (surpassing 0.20). |
| Woodward ND et al., 2005. [4] | Meta-Analysis | RCTs and prospective studies | I | 14 (SCZ) | SGAs (clozapine, olanzapine, quetiapine and risperidone) | The first analysis revealed that atypical antipsychotics are superior in improving overall cognitive function: the effect size for the Global Cognitive Index was significant (ES=0.24, Z=3.67, p<0.001). Specific improvements were observed in the learning (ES=0.24, Z=3.44, p<0.001) and processing speed domains (ES=0.21, Z=3.02, p<0.003). The second analysis extended the improvements to a broader range of cognitive domains and identified significant differences between treatments in attention (p<0.001; Qw=26.52, p>0.491; R2 = 0.46) and verbal fluency (p<0.002; Qw=25.18, p>0.912; R2 = 0.32). |
| Woodward ND et al., 2007. [5] | Meta-Analysis | RCTs | I | 14 (SCZ) | Haloperidol | The results indicate that overall cognitive performance improves while on haloperidol. In particular, the mean Global Cognitive Index effect size was significant (ES = 0.18, Z = 3.59, p < 0.001): specifically, the one derived from low dose studies was not significantly different from high dose studies (ES = 0.20 vs. 0.13, p < 0.548). |
| Riedel M et al., 2010.  [6] | Meta-Analysis | Double-blind and open label trials | I | 3  (129 SCZ) | Aripiprazole, olanzapine, quetiapine and risperidone. | A significant improvement in all cognitive domains was observed from baseline to week 8 (cognition index baseline -0.101, w4 0.006, w8 0.093, p<0.001). Regarding the antipsychotic treatment applied, quetiapine seemed to achieve the most favorable cognitive improvement. |
| Barry SJ et al., 2012. [7] | Systematic Review | Systematic Reviews, RCTs, and observational studies | I | 51 (SCZ) | FGAs and SGAs | Haloperidol may be effective for cognitive symptoms and comparable to other antipsychotics, although evidence is variable and weak. Compared with ziprasidone, olanzapine seems more effective at improving cognitive symptoms. There is no evidence of a difference between risperidone and clozapine or olanzapine in global neurocognitive score, while one  small study showed superiority of risperidone over haloperidol. |
| Désaméricq G et al., 2014. [8] | Network Meta-Analysis | RCTs | I | 9  (1540 SSD) | Antipsychotics | Quetiapine, olanzapine and risperidone had better effects on global cognitive score than amisulpride (p < 0.05) and haloperidol (p < 0.05). When memory tasks were considered, ziprasidone had better effect than amisulpride (0.28, 95% CI = 0.02-0.54) and haloperidol (0.32, 95% CI = 0.09-0.55). Quetiapine was better than other drugs (p < 0.001) on attention and processing speed tasks, followed by ziprasidone (p < 0.05) and olanzapine (p < 0.05). The effects of quetiapine, risperidone and olanzapine were better than those of amisulpride (p < 0.05) on executive functions. |
| Gabay AS et al., 2015. [9] | Meta-Analysis | Clinical Trials | I | 9  (1152 SCZ) | Antipsychotics | Overall, the study found a small, positive effect (Hedge's g = 0.13, 95% CI = 0.05 to 0.21, p = 0.002) of medication on facial affect processing. In a subgroup analysis this was statistically significant for atypical, but not typical, antipsychotics. Antipsychotic medications are poor at improving facial affect processing compared to reducing symptoms. |
| Nielsen RE et al., 2015. [10] | Meta-Analysis | RCTs | I | 37  (3526 SSD) | SGA | On cognitive composite score, sertindole was superior to clozapine (ES 0.87, 95% CI = 0.12-1.63), quetiapine (ES 0.75, 95% CI = 0.00-1.49 and FGAs (ES 0.89, 95% CI = 0.14-1.64). Analyses on separate cognitive domains showed clozapine (ES 0.37, 95% CI = 0.00 to 0.74) olanzapine (ES 0.31, 95%CI = 0.02 to 0.59), quetiapine (ES 0.34, 95% CI = 0.03 to 0.64), and FGAs (ES 0.51, 95% CI = 0.18 to 0.83) performed poorer on verbal working memory than ziprasidone, as well as FGAs performing poorer than risperidone (ES 0.31, 95% CI = 0.04 to 0.58. On executive function, sertindole performed better than clozapine (ES 0.82, 95% CI = 0.06 to 1.58), olanzapine (ES 0.81, 95% CI = 0.07 to 1.55), quetiapine (ES 0.76, 95% CI = 0.02 to 1.51), ziprasidone (ES 0.90, 95% CI = 0.14 to 1.67), and FGAs, (ES 0.83; 95% CI = 0.08 to 1.58). On processing speed, FGAs performed poorer than sertindole (ES 0.97, 95% CI = 0.02 to 1.91) and quetiapine (ES 0.36, 95% CI = 0.01 to 0.72). On long-term verbal working memory, clozapine performed poorer than olanzapine (ES 0.41, 95% CI = 0.06 to 0.76). On verbal fluency, FGAs performed poorer than olanzapine (ES 0.26, 95% CI = 0.01 to 0.50, and clozapine (ES 0.44, 95% CI = 0.06 to 0.81). Lastly, FGAs (ES 0.41, 95% CI = 0.04-0.78) and clozapine (ES 0.44, 95% CI = 0.05 to 0.83), performed poorer on visuospatial skill compared to olanzapine. |
| Schoretsanitis G et al., 2019. [11] | Systematic Review | Clinical trials | I | 19  (2317 various psychiatric disorders, mostly SCZ) | CLO/NCLO ratio | The association of the CLO/NCLO ratio impact on cognitive functioning is unclear: 4 cross-sectional studies reported a correlation between higher CLO/NCLO ratio and lower cognitive performance, whereas one post-hoc analysis of a randomized clinical trial yielded no significant correlation between the ratio and cognition. |
| Ohi K et al., 2020. [12] | Meta-Analysis | RCTs | I | 9  (1231 SCZ) | SGA | Compared with FGAs, SGAs have a superior effect on cognitive impairments, although the effect size is relatively small (Hedges' g = 0.25). The meta-analysis revealed nominally superior efficacy of SGAs compared with placebo in the treatment of cognitive impairments (I^2^ = 62.8, g = 0.22, p = 0.019). |
| Baldez DP et al., 2021. [13] | Meta-Analysis | RCTs | I | 54  (5866 SSD) | Antipsychotics | Favorable effects were observed for amisulpride, quetiapine, lurasidone, olanzapine, perphenazine, risperidone, sertindole, and ziprasidone, with small differences between molecules emerging in the different cognitive domains. Inferior effects were observed for remoxipride, clozapine and haloperidol, outperformed by placebo in most cognitive domains, as well as in the composite score evaluating global cognitive effects. |
| Haime Z et al., 2021. [14] | Systematic Review | Longitudinal or cross-sectional studies | I | 23  (773 HC, 1705 SCZ, 431 SSD, 21 MDD, 126 FEP, 18 psychosis, 28 BD, 113 early HD) | Antipsychotics and BDZ | Some studies found that antipsychotic treatment improves social cognition, while other studies found no effect on this cognitive skill. Neuroimaging findings suggest that medication may be affecting brain processes that have been found to be associated with social cognitive ability. |
| Kishimoto T et al., 2021.  [15] | Comparative Meta-Analysis | RCT, cohort studies, and pre-post studies | I | 137  (397 319 SSD) | LAIs and oral antipsychotics | Regarding outcomes related to cognitive function, LAIs were significantly more beneficial in two (10.5%) of 19 comparisons, not different in 16 (84.2%) comparisons, and less beneficial in one (5.3%) comparison. |

**BD** (Bipolar Disorder); **BDZ** (Benzodiazepines); **CI** (Confidence Interval); **CLO/NCLO ratio** (Clozapine/norclozapine ratio); **ES** (Effect Size); **FEP** (First Episode Psychosis); **FGAa** (First-Generation Antipsychotics); **HC** (Healthy Controls); **HD** (Huntingdon’s disease); **LAIs** (Long-Acting Injectable antipsychotics); **MDD** (Major Depressive Disorder); **RCTs** (Randomized Controlled Trials), **RR** (Relative Risk); **SCZ** (Schizophrenia); **SSD** (Schizophrenia Spectrum Disorders); **SGAs** (Second-Generation Antipsychotics).

**eTable 2. Pharmacological treatments – Other Pharmacological Treatments**

| **Authors** | **Type of Document** | **Types of Included Studies** | **Level of evidence** | **Number of Included Studies**  **(Number of participants and diagnoses)** | **Intervention** | **Main Findings** |
| --- | --- | --- | --- | --- | --- | --- |
| Chua WL et al., 2005.  [16] | Systematic Review | RCTs and open-label trial | I | 6 (SSD) | Estrogen | One included trial explored with several tests the impact on cognition of adjunctive estrogen and progesterone combined treatment compared to placebo. Results for visual retention tests showed statistically significant differences favoring the treatment group: total scores (n=8, WMD ‐3.5, 95% CI = ‐5.7 to ‐1.3) and error scores (n=8, WMD ‐12.0 95% CI = ‐17.6 to ‐6.4). Results for finger tapping test were not significant with wide confidence intervals, though the trend favored the treatment group. The mean endpoint scores also favored the treatment group on Verbal Fluency, Design Fluency, Auditory‐Verbal Learning Test, University of Pennsylvania Smell Test and Acuity‐smell sensitivity. |
| Tuominen HJ et al., 2005. [17] | Meta-Analysis | RCTs | I | 18  (343 SCZ) | Glutamatergic drugs | Available derived data on cognitive functioning do not indicate a significant effect of glycine or D-serine (N = 80, random effect model WMD = -2.79, 95% CI -6.17 to 0.60, p = 0.11). |
| Ferreri F et al., 2006. [18] | Systematic Review | RCTs | I | 14  (234 SSD) | ChEI adjunctive therapy (donepezil, rivastigmine, and galantamine) | This review highlights the insufficiency of evidence to prove ChEI efficacy in the treatment of cognitive dysfunctions in schizophrenia. There are findings that support the idea that ChEI may have a beneficial effect in enhancing cognitive functions. However, most of the double-blind controlled trials suggest no efficacy of this add-on strategy. |
| Premkumar TS et al., 2006.  [19] | Systematic Review | RCTs | I | 2  (329 SCZ) | Lamotrigine | Most cognitive measures showed no differences (n=329, 2 RCTs, RR not attaining BACS composite score of 0.5 1.10, CI 0.59 to 2.04) between the lamotrigine and the placebo group. |
| Tuominen HJ et al., 2006.  [20] | Systematic Review | RCTs | I | 18  (358 SSD) | Glutamatergic drugs (glycine, D-serine, D-cycloserine, or ampakine CX516) + antipsychotics | Available data did not indicate a statistically significant effect of glycine or D-serine on cognitive functioning. Specifically, none of the 34 participants in either glycine or control group reached 50% recovery in two trials, and also using the less conservative cut off point of 20% did not produce any statistically significant difference between glycine and the control group (n=34, 2 RCTs, RR 0.72, 95% CI = 0.4 to 1.3). Also, no statistically significant difference when participants were allocated to either glycine or D-serine, compared with the control group (n=80, 4 RCTs, WMD -2.79 95% CI = -6.2 to 0.6) was found considering average endpoint score (PANSS-cognitive). |
| Chouinard S et al., 2007. [21] | Meta-Analysis | RCTs, crossover and open trials | I | 12  (445 SCZ) | ChEI (donepezil, rivastigmine, and galantamine) | The results reveal a small improvement in attention after ChEI medication in schizophrenia (effect estimate comparing before and after add-on treatment with ChEI: ES 0.261, p=0.006, 95% CI = 0.074 to 0.448). As a final consideration it was stated that no clear conclusion can yet be reached concerning the cognitive-enhancing effects of ChEI considering the small number of studies available. |
| Stip E et al., 2007. [22] | Meta-Analysis | RCTs, crossover and open trials | I | 10  (447 SCZ) | ChEI (donepezil, rivastigmine, and galantamine) | This meta-analysis provides no clear evidence on whether ChEIs should be prescribed for memory enhancement in patients with schizophrenia.  In particular, the analysis broken down by ChEI found no significant effect: 5 studies of donepezil (ES = −0.352, p = 0.094), 2 studies of rivastigmine (ES = 0.383, p = 0.249), and 1 study of galantamine yielded heterogeneous and non-significant results. |
| Saavedra-Velez C et al., 2009. [23] | Systematic Review | 5 RCTs and 1 open-label pilot study | I | 6  (153 SCZ) | Modafinil | Three of 6 reviewed studies showed that modafinil may improve short-term memory, attention, and the ability to shift mental sets. Two neuroimaging studies identified functional correlates in areas associated with working memory functions. |
| Ribeiz SR et al., 2010. [24] | Meta-Analysis | RCTs | I | 13  (564 SSD) | ChEI (donepezil, rivastigmine, and galantamine) | The reviewed studies suggest that specific cognitive deficits (memory, and the motor speed and attention part of executive function) of patients with schizophrenia and schizoaffective disorder are responsive to rivastigmine, donepezil and galantamine as adjunctive therapy. |
| Singh J et al., 2012.  [25] | Systematic Review | RCTs | I | 7  (187 SSD) | ChEIs | The acetylcholinesterase inhibitor plus antipsychotic showed benefit over antipsychotic and placebo in attention, (1 RCT, n = 73, MD 1.20, 95% = CI 0.14 to 2.26), visual memory (2 RCTs, n = 48, MD 1.90, 95% CI = 0.52 to 3.28), verbal memory and language (3 RCTs, n = 42, MD 3.46, 95% CI = 0.67 to 6.26) and executive functioning (1 RCT, n = 24, MD 17.10, 95% CI = 0.70 to 33.50). |
| Choi KH et al., 2013. [26] | Meta-Analysis | RCTs | I | 26  (1104 SSD) | Medications targeted at cholinergic, glutamatergic or serotonergic receptors. | Medications targeted at the cholinergic receptor class produced marginal improvements in verbal learning and memory (d = 0.23, P = 0.06) and, in particular, donepezil produced a moderate effect (d = 0.58) on spatial learning and memory. |
| Kishi T et al., 2013.  [27] | Meta-Analysis | RCTs | I | 8  (347 SSD + 59 BD) | NMDAR-ANTs | NMDAR-ANTs as adjunctive therapy may improve cognitive function in patients with schizophrenia, specifically NMDAR-ANTs adjunctive therapy was superior to placebo in MMSE scores in only schizophrenia/schizoaffective disorder (SMD = −0.77, 95% CI = −1.27 to −0.28, p = 0.002, I^2^ = 0%, N = 3, n = 71). |
| Scoriels L et al., 2013.  [28] | Systematic Review | RCTs | I | 9  (228 SSD) | Modafinil | A review of its effects in schizophrenia suggests that modafinil facilitates cognitive functions, with pro-mnemonic effects and problem-solving improvements. |
| Vernon JA et al., 2014.  [29] | Meta-Analysis | RCTs | I | 11  (568 SCZ) | Antidepressants (mirtazapine, citalopram, fluvoxamine, duloxetine, mianserin, bupropion and reboxetine) | Adjunctive antidepressants do not demonstrate clinically significant effects on cognition in schizophrenia patients. Statistically significant, but clinically negligible, advantages were found for pooled antidepressants compared to placebo in executive function (Hedges' g = 0.17, p = 0.02) and in the cognitive composite score (Hedges' g = 0.095, p = 0.012). |
| Andrade C et al., 2015.  [30] | Meta-Analysis | RCTs | I | 8  (372 SCZ) | Modafinil or armodafinil | Although data were limited, cognition did not differ significantly between modafinil/armodafinil and placebo groups (mean difference, −0.14; 95% CI, −0.62 to 0.34; p = 0.57). |
| Heringa SM et al., 2015.  [31] | Meta-Analysis | RCTs | I | 12  (724 SSD) | Estrogens, SERMs, Testosterone, DHEA, Pregnenolone and Oxytocin | Results for cognition (k=12) were too diverse for meta-analyses, and inspection of these data showed no consistent benefit. |
| Iseger TA et al., 2015.  [32] | Systematic Review | Clinical trials | I | 29  (SCZ) | CBD | Results suggest that CBD could be helpful in counteracting psychotic symptoms and cognitive impairment associated with cannabis use as well as with acute THC administration. |
| Iwata Y et al., 2015. [33] | Meta-Analysis | RCTs | I | 17  (1391 SSD) | Glutamate positive modulators | Glutamate positive modulators were not superior to placebo in terms of overall cognitive function (SMD=0.08, 95% confidence interval=-0.06 to 0.23) (11 studies, n=858) nor each of eight cognitive domains (SMDs=-0.03 to 0.11) (n=367-940) in this population. |
| Rowe AR et al., 2015. [34] | Systematic Review | RCTs | I | 19  (951 SSD) | Nicotinic modifying compounds: α7 and α4ß2 receptor agonists, and positive allosteric modifying drugs. | Findings for α4ß2 receptor agonists were extremely limited, with no significant cognitive improvements evidenced; the PAM data were similarly disappointing, with the only positive outcome for galantamine in a study with a very small sample size. The α7 receptor agonists produced slightly stronger results. |
| Terevnikov V et al., 2015.  [35] | Systematic Review | RCTs | I | 4  (139 SSD) | Antidepressants | Citalopram did not improve cognition in schizophrenia, whereas Bupropion improved attention in the same patients. Also, Mianserin and Mirtazapine improved neurocognition in FGA-treated schizophrenia patients. |
| Magalhães PV et al., 2016.  [36] | Systematic Review | RCTs | I | 22  (2041 SSD) | Antioxidant treatments | No clinically important change in overall cognitive functioning was found with ginkgo biloba, NAC, allopurinol, DHEA, vitamin C, vitamin E or selegiline. |
| Zheng W et al., 2016.  [37] | Meta-Analysis | RCTs | I | 12  (1117 SSD) | HupA | This review suggests that adjunctive HupA is an effective choice for improving cognitive function for patients with schizophrenia spectrum disorders. HupA outperformed comparators on the following outcome measures: the WMS-R including memory quotient (WMD: 10.59; 95% CI: 5.65, 15.53; p < 0.0001); WAIS-R including verbal IQ, performance IQ, and full IQ (WMD: 3.97 to 5.66; 95%CI: 0.20, 8.58; p = 0.01 to 0.00001); WCST including response administer and non-perseverative errors (WMD: -12.79 to -12.29; 95%CI: -23.70, -0.88; p = 0.03 to 0.003). |
| Bürkner PC et al., 2017.  [38] | Meta-Analysis | RCTs | I | 12  (273 SSD) | Intranasal Oxytocin | Intranasal Oxytocin did not improve social cognition (SMD = 0.07, 95% CI = -0.06 to 0.17) or neurocognition (SMD = 0.12, 95% CI = -0.12 to 0.34). Intranasal Oxytocin had a significantly larger effect on high-level social cognition (ie, mentalizing and theory of mind) compared to low-level social cognition (ie, social cue perception). |
| Correll CU et al., 2017.  [39] | Meta-Analysis | RCTs | I | 381  (19 833 SSD) | Pharmacologic Co-treatment Strategies Added to Antipsychotic Monotherapy | Cognitive disfunctions were not improved with combination strategies, except for n-methyl-d-aspartate receptor antagonists (SMD, −0.77, 95% CI = −1.26 to −0.28) and pooled antidepressants (SMD −0.10, 95% CI, −0.17 to −0.02). |
| Di Iorio G et al., 2017. [40] | Systematic Review | Open label or double-blind trials, prospective or retrospective observational studies, and case reports | I | 10 (SCZ) | Memantine | Memantine therapy in patients with schizophrenia seems to improve mainly negative symptoms while positive symptoms and cognitive dysfunctions did not improve significantly. |
| Jin Y et al., 2017. [41] | Meta-Analysis | Randomized double-blind placebo-controlled trials | I | 8  (1438 SCZ) | α7-nAChR agonists | There was no statistically significant difference favoring α7-nicotinic agonists in terms of overall cognitive function (SMD=-0.10[-0.46, 0.25], I^2^=88%) in patients with schizophrenia according to MCCB overall scores. |
| Kishi T et al., 2017. [42] | Meta-Analysis | RCTs | I | 8  (448 SCZ) | Memantine | Memantine add-on treatment was superior to placebo for ameliorating MMSE score (MD= -3.07, 95% CIs= -4.46 to -1.69, p<0.0001). |
| Lewis AS et al., 2017. [43] | Meta-Analysis | Clinical Trials | I | 18  (2670 SCZ or AD) | α7-nAChR agonists | Cognitive outcomes were standardized, and random-effects meta-analyses revealed no statistically significant effects of α7 nAChR agonists on overall cognition or any of eight cognitive subdomains when all doses were included (Range of all cognitive outcomes: Cohen's d=-0.077 to 0.12, negative favoring drug). In contrast, analysis of 29 rodent studies testing the same α7 agonists revealed large effect sizes in multiple commonly used preclinical behavioral tests of cognition (Range: d=-1.18 to - 0.73). Results suggest that targeting the α7 nAChR with agonists is not a robust treatment for cognitive dysfunction in SCZ or AD and necessitate a better understanding of the translational gap for therapeutics targeting the α7 nAChR. |
| Solmi M et al., 2017. [44] | Meta-Analysis | RCTs, open non-randomized trials and case series | I | 6  (413 SSD) | Minocycline | Attention (p=0.47), memory (p=0.52), and motor speed processing (p=0.50) did not significantly differ from placebo, before execution of a trim-and-fill procedure. |
| Kishi T et al., 2018.  [45] | Meta-Analysis | RCTs | I | 37  (1574 SSD) | Anti-Dementia Drugs  (Donepezil, galantamine, rivastigmine and memantine) | Pooled anti-dementia drugs plus antipsychotics treatments were superior to placebo plus antipsychotics in improving MMSE scores (7 studies, 225 patients, SMD = -0.79, 95% CI = -1.23 to -0.34, p= 0.0006). However, it was not superior to placebo plus antipsychotics in improving composite cognitive score. |
| Matthews PRL et al., 2018.  [46] | Systematic Review | RCTs | I | 16  (919 SSD) | NRIs | Significant response or improvement in cognitive functioning data were not reported. Average composite cognitive scores showed no difference between NRIs and placebo (4 RCTs, n = 180; SMD 0.04, 95% CI -0.28 to 0.36; low-quality evidence). |
| Santos B et al., 2018.  [47] | Meta-Analysis | RCTs | I | 9  (348 SSD) | ChEIs | Six randomized controlled trials ( n=219) presented evidence that acetylcholinesterase inhibitors improve speed of processing (SMD -0.52, 95% CI -0.79 to -0.25; p = 0.0002). However, eight randomized controlled trials (n=252) did not find placebo was better than acetylcholinesterase inhibitors in the attention domain (-0.43, 95%CI -0.72 to -0.1, p = 0.005) and eight randomized controlled trials (n=273) did not find differences in the working memory (-0.14, 95% CI -0.51 to 0.24, p = 0.47). |
| Sinkeviciute I et al., 2018. [48] | Meta-Analysis | Clinical trials | I | 93  (5630 SCZ) | Drugs acting on glutamatergic; cholinergic; serotonergic; dopaminergic; GABA-ergic; noradrenergic; miscellaneous systems | Cognitive enhancers were not superior to placebo in improving cognitive functions. When analyzing each neurotransmitter system separately, agents acting predominantly on the glutamatergic system showed a small significant effect on overall cognition (k = 29, Hedges' g = 0.19, p = 0.01), as well as on working memory (k = 20, Hedges' g = 0.13, p = 0.04). A sub-analysis of cholinesterase inhibitors showed a small effect on working memory (k = 6, Hedges' g = 0.26, p = 0.03). |
| Soria V et al., 2018.  [49] | Systematic Review | RCTs | I | 15  (895 SCZ, BD, MDD) | Drugs targeting the HPA axis (mifepristone, ketoconazole, dehydroepiandrosterone) and sex steroids (raloxifene, pregnenolone) | Among sex steroids, raloxifene and pregnenolone show the most promising results as cognitive enhancers in patients with schizophrenia |
| Tan BL et al., 2018. [50] | Systematic Review | RCTs | I | 9  (280 SSD) | Oxytocin | Four studies reported significant effects on high level social cognition (comprehension of indirectly expressed emotions/thoughts based on complex integration of social contextual information) but not on low-level social cognition (emotional perception and social cue detection). This was in contrast with two earlier studies which obtained treatment effects on emotional recognition. In one study oxytocin was found to yield benefits in empathic accuracy and emotion identification. |
| Zheng W et al., 2018.  [51] | Meta-Analysis | RCTs | I | 5  (242 SCZ) | Azapirones (buspirone and tandospirone) | Only 2 RCTs found the superiority of buspirone in improving attention/speeded motor performance, verbal and performance intelligence. Other RCTs did not show significant group differences in terms of cognitive functions. In conclusion, adjunctive buspirone may be associated with improvement in cognitive deficits in schizophrenia but, due to the preliminary nature of this meta-analysis, larger sample size and higher quality RCTs are needed to confirm these finding. |
| Chang CH et al., 2019. [52] | Meta-Analysis | RCTs | I | 25  (1951 SSD) | NMDA-receptor-enhancing agents | NMDA-receptor-enhancing agents had no superior effect compared with to placebo on overall cognitive function (SMD = 0.068, CI = -0.056 to 0.193, p = 0.283). However, subgroup analysis suggested that NMDA-receptor-enhancing agents may benefit young patients with schizophrenia, and N-acetyl cysteine may have an effect on WM (SMD = 0.679, 95% CI = 0.397 to 0.961, p < 0.001). |
| Cho M et al., 2019.  [53] | Meta-Analysis | RCTs | I | 14  (895 SSD) | Aspirin, celecoxib, omega-3 fatty acids, estrogen, SERMs, pregnenolone, N-acetylcysteine, minocycline, davunetide and erythropoietin. | Significant cognitive benefits were found for minocycline (*k* = 10, *g* = 0.21, 95% CI =0.04 to 0.38) and pregnenolone (*k* = 20, *g* = 0.19, 95% CI = 0.08 to 0.29). There was no significant cognitive improvement when the effects of omega-3, estrogens, SERM, aspirin, davunetide and erythropoietin were pooled. |
| Ortiz-Orendain J et al., 2019.  [54] | Meta-Analysis | RCTs | I | 11  (422 SDD) | Modafinil | Only 1 RCT (n = 48) explicitly explored the effect of modafinil on cognitive function (using the MCCB): in this study there was no clear difference in scores between modafinil and placebo treatment groups (MD -3.10, 95% CI = -10.9 to 4.7). |
| Solmi M et al., 2019.  [55] | Meta-Analysis | RCTs | I | 22  (644 SSD) | Atomoxetine, amphetamines and methylphenidate | Attention, processing speed, working memory, problem solving, and executive functions showed from no to some improvement with atomoxetine or amphetamines. Meta-analysis did not confirm any effect of stimulants in any cognitive domain, apart from atomoxetine improving problem solving (k=2, SMD=0.73, 95% CI=0.10-1.36, p=0.02, I2=0%), and trending toward significant improvement in executive functions with amphetamines (k=2, SMD=0.80, 95% CI=-1.68 to +0.08, p=0.08, I2=66%). |
| Zheng W et al., 2019.  [56] | Meta-Analysis | RCTs | I | 4  (280 SCZ) | Ondansetron | Although 80% RCTs examined the effects of ondansetron on cognitive functions, different measures were applied rendering the data unsuitable for meta-analysis. Two RCTs found ondansetron superior over placebo in the cognitive items of the PANSS. Ondansetron could significantly improve visual memory and object assembly and comprehension. The effect of ondansetron on cognitive impairment in schizophrenia needs to be further explored in large-scale RCTs. |
| Zheng W et al., 2019.  [57] | Meta-Analysis | RCTs | I | 15  (512 SCZ, 319 BP,157 MDD) | Memantine | Memantine outperformed the comparator in improving cognitive performance in schizophrenia (SMD = 1.07, 95% CI = 0.53 to 1.61; P < 0.0001, I^2^ = 29%). |
| Chang CH et al., 2020.  [58] | Meta-Analysis | RCTs | I | 4  (186 SCZ) | Sarcosine (N-methylglycine) | For overall cognitive functions, sarcosine showed a positive but insignificant effect (SMD = 0.27, 95% CI = -0.06 to 0.60, *p* = 0.10). |
| Dondé C et al., 2020.  [59] | Systematic Review | RCTs | I | 19  (621 SSD) | Acute nicotine administration | The findings highlight that a single dose of nicotine can improve a range of cognitive functions in schizophrenia subjects, such as attention, working memory, and executive functions, with attention being the most responsive domain. |
| Jeppesen R et al., 2020.  [60] | Meta-Analysis | RCTs | I | 70  (4104 SSD) | Anti-inflammatory agents | Analyses showed superior effect of anti-inflammatory add-on on working memory compared to placebo (SMD=0.21). No significant effect was found for composite scores, executive functions, attention, processing speed or verbal fluency. |
| Koola MM et al., 2020. [61] | Meta-Analysis | RCTs | I | 6  (226 SCZ) | Galantamine | Cognitive impairments significantly improved with galantamine compared to placebo, with a small Hedges' g effect size of 0.233. |
| Kopelli E et al., 2020.  [62] | Meta-Analysis | RCTs | I | 3  (166 SDD) | CBD oil | In one study, CBD oil was compared with amisulpride as monotherapy treatment, but no data were available for cognition. The other two studies estimated the effects of CBD oil as add-on treatment compared to placebo: no significant difference was found in cognition (SMD = 0.09, 95% CI = −0.27 to 0.45, 2 RCTs, N = 121). |
| Tanzer T et al., 2020. [63] | Meta-Analysis | RCTs | I | 4  (339 SSD) | Varenicline | Varenicline was not superior to placebo for overall cognition (SMD = -0.022, 95% CI = -0.154 to 0.110, Z = -0.333, p = 0.739), attention (SMD = -0.047, 95% CI = -0.199 to 0.104, Z = -0.613, p = 0.540), executive function (SMD = -0.060, 95% CI = -0.469 to 0.348, Z =- 0.290, p = 0.772) or processing speed (SMD = 0.038, 95% CI = -0.232 to 0.308, Z = 0.279, p = 0.780). |
| Yolland CO et al., 2020.[64] | Meta-Analysis | RCTs | I | 5  (275 SCZ or FEP) | N-acetylcysteine | The cognitive domain of working memory improved with N-acetylcysteine supplementation (SMD = 0.56, p = 0.005). |
| Zheng W et al., 2020. [65] | Meta-Analysis | RCTs | I | 5  (284 SCZ) | Adjunctive fluvoxamine | Only 2 studies examined the effects of adjunctive fluvoxamine on cognitive functions. Adjunctive fluvoxamine was superior to placebo in improving cognitive functions measured with the Spatial Working Memory (including both working visuospatial memory and strategy use) of the Cambridge Neuropsychological Test Automated Battery11 and the WMS10. No significant differences for other dimensions of the Cambridge Neuropsychological Test Automated Battery were found between the two groups. |
| Ahmed S et al., 2021. [66] | Systematic Review | RCTs | I | 11  (306 SSD) | THC or CBD | There is insufficient evidence to define an effect of THC or CBD on symptoms, cognition, and neuroimaging measures of brain function in schizophrenia. |
| Kuppili PP et al., 2021. [67] | Meta-Analysis | RCTs | I | 4  (200 SCZ) | D-Cycloserine | D-Cycloserine did not exhibit significant efficacy in treating cognitive dysfunction in schizophrenia at either study-defined endpoint (4-36 weeks) or at four weeks (early outcome) (pooled SMD = − 0.05, 95% CI= − 0.91 to 0.81). |
| Recio-Barbero M et al., 2021. [68] | Systematic Review | RCTs | I | 13  (1392 SSD) | Antipsychotics + α7 nAChR agonists | No differences were found in any of the cognitive domains assessed in four RCTs. In contrast, nine RCTs presented a small effect in support of α7 nAChR agonists for negative symptoms (SMD = -0.28, 95% CI = -0.56 to -0.00, p = 0.050), even though the confidence to support this evidence is low according to the GRADE system. |
| Seetharam JC et al., 2022. [69] | Meta-Analysis | RCTs | I | 4 (SCZ) | Sodium benzoate 1000–2000 mg per day as adjunctive therapy | Add-on sodium benzoate can improve positive symptoms of schizophrenia significantly (MD: -1.87, 95% CI = -3.25 to -0.48; p = 0.008) but had no significant favourable effect on negative symptoms (p = 0.84), general psychopathology (p = 0.49), and total PANSS score (p = 0.19) over the control. There was no significant improvement in GAF (p = 0.43), CGI (p = 0.58), cognitive function (p = 0.46) and quality of life (p = 0.73). Extrapyramidal symptoms were significantly higher (MD: 0.39, 95% CI:0.19 to 0.60; p = 0.0002) in the sodium benzoate group in comparison to the control group; however, there was no significant difference in respect to  other adverse events. |

**AD** (Alzheimer’s Disease); (Brief Assessment of Cognition in Schizophrenia); **BD** (Bipolar Disorder); **CBD** (cannabidiol); **CGI** (Clinical Global Impressions); **ChEI** (Cholinesterase inhibitors); **CI** (Confidence Interval); **DHA** (Docosahexaenoic acid); **DHEA** (Dehydroepiandrosterone); **ES** (Effect Size); **FEP** (First Episode Psychosis); **GABA** (Gamma-aminobutiric Acid); **GAF** (Global Assessment of Functioning); **HPA** (Hypothalamic-Pituitary-Adrenal); **HupA** (HuperzineA); **MCCB** (MATRICS Consensus Cognitive Battery); **MD** (Mean Difference); **MDD** (Major Depressive Disorder); **MMSE** (Mini Mental Status Examination); **NAC** (N-Acetyl Cysteine); **nAChR** (nicotinic Acetylcholine Receptor); **NMDAR-ANTs** (NMDA receptor antagonists); **NRIs** (selective Noradrenaline Reuptake Inhibitors); **NMDA** (N-methyl-D-aspartate); **PAMs** (Positive Allosteric Modulators**); PANSS** (Positive And Negative Symptoms Scale); **RCTs** (Randomized Controlled Trials), **RR** (Relative Risk); **SCZ** (schizophrenia); **SSD** (schizophrenia-spectrum disorders); **SERMs** (Selective Estrogen Receptor Modulators); **SMD** (Standardized Mean Difference); **THC** (tetrahydrocannabinol); **WAIS-R** (Wechsler Adult Intelligence Scale-Revised); **WCST** (Wisconsin Card Sorting Test); **WMS-R** (Wechsler Memory Scale-Revised); **WM** (Working Memory); **WMD** (Weighted Mean Difference).

**eTable 3. Psychosocial interventions - Cognitive Remediation**

| **Authors** | **Type of Document** | **Types of Included Studies** | **Level of evidence** | **Number of Included Studies**  **(Number of participants and diagnoses)** | **Intervention** | **Main Findings** |
| --- | --- | --- | --- | --- | --- | --- |
| Roder V et al., 2006  [70] | Quantitative review | RCTs | I | 7  (362 SCZ) | IPT | Positive mean effect sizes favoring IPT over control groups (placebo-attention conditions, standard care) were found for all dependent variables, including symptoms, psychosocial functioning, and neurocognition. Moreover, the superiority of IPT continued to increase during an average follow-up period of 8.1 months. |
| McGurk SR et al., 2007.[71] | Meta-analysis | RCTs | I | 26  (1151 SSD) | CR | Cognitive remediation was associated with significant improvements with a medium effect size for cognitive performance (0.41).  Six studies also reported cognitive data at follow-up: for these studies, the average effect size at posttreatment was 0.56 (t=4.8, p<0.001, 95% CI =0 .33 to 0.79), and at follow-up, it was 0.66 (t=5.7, p<0.001, 95% CI = 0.43 to 0.89) Similar to the results at posttreatment, cognitive remediation was associated with improved overall cognitive performance an average of 8 months later. Hedges’s Q was significant for only one cognitive domain, verbal learning and memory, indicating significant heterogeneity in effect sizes to evaluate the effects of moderators. |
| Roder V et al., 2011. [72] | Meta-analysis | RCTs + non controlled trials | I | 35  (1575 SCZ) | IPT | The strongest effect was found in social cognition (ES = 0.70), but the Q value of the ES for social cognitive change suggests heterogeneous effects across studies. Comparing the IPT effects with those of the 2 control conditions (placebo-attention condition -unspecific group activities- and treatment as usual), significant effects favoring IPT were evident in neurocognition, social cognition, and functional outcome (QB > 13.7, p <0.01). |
| Wykes T et al., 2011. [73] | Meta-analysis | RCTs | I | 40  (2104 SCZ) | CR | This meta-analysis found an overall positive effect of cognitive rehabilitation of 0.45 (95% CI = 0.31 to 0.59). Also, the effects of treatment on global cognition were durable (N= 11, ES = 0.43, 95% CI = 0.18 to 0.67). |
| Kurtz MM et al., 2012. [74] | Meta-analysis | Clinical trials | I | 19  (692 SSD) | SCT | With respect to social cognitive measures, weighted effect-size analysis revealed that there were moderate-large effects of social cognitive training procedures (identification, d = 0.71 and discrimination, d = 1.01) and small-moderate effects of training on ToM (d = 0.46), while effects on social cue perception and attributional style were not significant. |
| Kluwe-Schiavon B et al., 2013. [75] | Systematic review | RCTs | I | 30  (1981 SSD) | CR | The reviewed articles corroborate the literature pointing that CR could be a promising therapeutic option for cognitive deficits in schizophrenia.  A proportion of 23% of studies scored higher than 4 points in JADAD scale, 40% scored 3 points, 33% scored 2 points and one study scored only 1 point. |
| Mueller DR et al., 2013. [76] | Meta-analysis | RCTs | I | 15  (632 SCZ) | IPT | Significant medium to large effect sizes (ES) were evident for IPT independent of age on the global cognitive score (mean score of all cognitive variables), on neurocognition, social cognition, social functioning, psychopathology, and the global therapy effect (mean of all variables). Investigating age-related effects in IPT showed significantly stronger effects in older IPT patients compared with younger IPT patients on executive functioning (Qb = 7.30, p < 0.01) and on social/emotion perception (Qb = 17.85, p < 0.01), but not on memory and attention (Qb ≤ 0.65, p > 0.42). |
| Paquin K et al., 2014.  [77] | Systematic review | Clinical trials | I | 99  (SSD) | CR; SCIT; CRT; CBT | In neurocognition, drill and practice training is used more frequently and with a variety of different procedures such as auditory training or target discrimination. Tailoring the training to specifically address precise deficits might be one of the key benefits of drill and practice training. However, from the evaluated studies, drill and strategy training was more easily generalized to all neurocognitive deficits. |
| Thorsen AL et al., 2014.  [78] | Systematic review | Clinical trials | I | 13  (494 SCZ and HC) | CRT; AT; CET; EST; CBSST; social training; Cogpack | The reviewed studies indicate that CRT affects several brain regions and circuits, including prefrontal, parietal, and limbic areas, both in terms of activity and structure. Changes in prefrontal areas are the most reported finding, fitting to previous evidence of dysfunction in this region. |
| Revell ER et al., 2015. [79] | Meta-analysis | RCTs | I | 11  (615 FEP or early SCZ) | CR | No significant positive effect of CR was observed on global cognition (ES = 0.13, 95% CI = 0.04 to 0.31; p = 0.14) in the main analysis. However, a significant positive effect was observed in a sensitivity analyses conducted removing clear outliers (ES =0.19, 95% CI = 0.00 to 0.38, p < 0.05).  A significant positive effect of CR was also observed in the verbal learning and memory domain (ES = 0.23, 95% CI = 0.01 to 0.46, p = 0.046) |
| Isaac C et al., 2016.  [80] | Systematic review | RCTs | I | 19  (455 SSD) | CR | Cognitive remediation therapy seems to provide a neurobiological enhancing effect in schizophrenia. After therapy, increased activations are observed in various brain regions mainly in frontal (especially prefrontal), occipital and anterior cingulate regions during working memory and executive tasks. Several studies provide evidence of an improved functional connectivity after cognitive training, suggesting a neuroplastic effect of therapy through mechanisms of functional reorganization. Neurocognitive and social-cognitive training may have a cumulative effect on neural networks involved in social cognition. |
| Wei YY et al., 2016. [81] | Meta-analysis | Clinical trials | I | 9  (204 SCZ) | CRT | The current findings suggest that CRT might improve the cognition of schizophrenia patients by increasing activations of the frontal and parietal lobe. |
| Grant N et al., 2017. [82] | Systematic review | RCTs | I | 32  (1440 SSD) | SCIT | The results suggest that both targeted and broad-based interventions are effective, particularly for theory of mind and affect recognition, but they do not seem to provide significant benefits for attributional style. Social perception improved in the majority of studies. |
| Morin L et al., 2017. [83] | Systematic review | Review articles and meta-analyses | I | 80  (SSD) | CR, psychoeducation, social skills training, and cognitive therapy | According to results, cognitive remediation has been found to be effective in reducing the impact of cognitive impairment, social skills and, to a lesser extent, in reducing negative symptoms. |
| Tan B-L et al., 2018. [50] | Systematic review | Clinical trials | I | 61  (SSD) | IPT, CET, SCIT, SCST, MSCT, online training program (SocialVille), AS, METT, TAR, ETIT, TOMI, SoCog-MSRT, MEB, SCTP, SCET, oxytocin, tDCS | Various types of social cognitive interventions have produced positive outcomes either on specific social cognitive domains or across domains. |
| Vass E et al., 2018. [84] | Systematic review | RCTs | I | 17  (681 SSD) | TOMI; other techniques targeting other areas of social cognition | Targeted ToM intervention produced more improvement in ToM tasks, while data regarding non-ToM interventions showed contradictory results with limited effects on ToM. |
| Kambeitz-Ilankovic L et al., 2019. [85] | Meta-analysis | Clinical Trials | I | 67  (4067 SSD) | CR with supplementary human guidance | There was a moderate effect of CR on cognitive outcomes (g = 0.28). Within the cognitive domains, there were significant effects of CR on attention (g = 0.23), verbal memory (g = 0.21), social cognition (g = 0.18), working memory (g = 0.27), processing speed (g = 0.22), reasoning (g = 0.25), and cognitive global (g = 0.26). No significant effect was present for visual memory. |
| Prikken M et al., 2019. [86] | Meta-analysis | Clinical trials | I | 24  (1262 SSD) | Computerized cognitive drill and practice training | Compared to a control condition, patients receiving computerized cognitive drill and practice training showed significantly more improvement on attention (ES = 0.31, p = 0.001) and working memory (ES = 0.38, p b 0.001). Small, marginally significant effect sizes were found for processing speed, verbal and visual learning and memory, and verbal fluency. However, significant effects on functional outcomes  and social cognition were absent. |
| Cella M et al., 2020. [87] | Meta-analysis | RCTs | I | 20  (1509 SSD) | CR | Results from random-effect models suggested that CR was effective in improving processing speed (*g* = 0.48), memory (*g* = 0.48) and working memory (*g* = 0.56). While there was an indication of improvements in the levels of vocational, social and global functioning, these were less reliable. |
| Datta SS et al., 2020. [88] | Systematic review | RCTs | I | 7  (319 psychosis) | CRT; GPT; CRP + PTP; PE + MFT; Nonstructured Group Therapy | Compared to TAU, CRT may have a positive effect on cognitive functioning, some data suggests TAU may have positive effect on mental state. GPT may have a positive effect on global state. |
| Gandara V et al., 2020. [89] | Systematic review | Clinical Trials | I | 14  (238 SCZ) | Neurofeedback | NF treatment appears to influence neural processing, connectivity and metabolism in the brain, as shown by changes in pre/post scalp electrical activity and neuroimaging studies. |
| Jahn FS et al., 2021.  [90] | Systematic review | RCTs | I | 3  (44 SCZ, ADHD) | Cognitive training with fully immersive virtual reality | The cognitive domains which improved significantly after VR training were theory of mind, visual working memory and executive functions. |
| Lejeune JA et al., 2021. [91] | Meta-analysis | RCTs | I | 73  (4594 SSD) | CR | The overall effect of CR on cognition was 0.29 (95% CI= 0.12 to 0.45), indicating a small effect on global cognition. Within the standard cognitive domains of the MCCB, there were significant small-to-moderate effects of CR on verbal learning (k = 61, g = 0.33), working memory (k = 62, g = 0.32), attention (k = 35, g = 0.28), reasoning and problem-solving (k = 50, g = .27), processing speed (k = 52, g = 0.20), and visual learning (k = 33, g = 0.19). Effects on social cognition were small and marginally significant (k = 17, g = 0.12, 95% CI = 0.00 to 0.24). |
| Vita A et al., 2021.  [92] | Meta-analysis | RCTs | I | 130  (8851 SSD) | CR | CR was effective on cognition (d = 0.29, 95% CI =0.24 to 0.34) and functioning (d = 0.22, 95% CI, 0.16 to 0.29]). An active and trained therapist (cognition: p = 0.04; functioning p = 0.04), structured development of cognitive strategies (cognition: p = 0.002; functioning: p = 0.004), and integration with psychosocial rehabilitation were crucial ingredients of efficacy. Patients with fewer years of education (global cognition: coefficient, −0.055, 95% CI = −0.103 to −0.006; p = 0.03; global functioning: coefficient, −0.061, 95% CI = −0.112 to −0.011, p = 0.02), lower premorbid IQ (global functioning: coefficient, −0.013, 95% CI = −0.025 to −0.001, p = 0.04), and higher baseline symptom severity (global cognition: coefficient 0.006, 95% CI = 0.002 to 0.010, p = 0.005) emerged as optimal candidates to CR. |
| Yeo H et al., 2022. [93] | Meta analysis | RCTs | I | 42  (1868 SSD) | SCT | SCT in this meta-analysis has been found to be effective for social perception (estimated effect size: g = 0.46, SE = 0.15, 95% CI = 0.13to 0.80, p < .05), emotional recognition (effect size: g = 0.55, SE = 0.10, 95% CI = 0.34 to 0.75, p < .001), mental state attribution tasks (effect size: g = 0.36, SE = 0.11, 95% CI = 0.13 to 0.59, p < .01). |

**ADHD** (Attention Deficit Hyperactivity Disorder); **AS** (Attentional Shaping); **AT** (Auditory based Training); **CBSST** (Cognitive Behavioural Social Skills Training); **CBT** (cognitive behavioral therapy); CCPT (Conners Continuous Performance Task); **CET** (Cognitive Enhancement Therapy); **CI** (Confidence Interval); **CR** (Cognitive Remediation); **CRP** (Cognitive Remediation Programme); **CRT** (Cognitive Remediation Therapy); **ES** (Effect Size); **EST** (Enriched Supportive Therapy); **ETIT** (Emotion and ToM Imitation); **GPT** (Group Psychosocial Therapy); **HC** (Healthy Controls); **IQ** (Intelligence Quotient); **IPT** (Integrated Psychological Therapy); **MCCB** (MATRICS Consensus Cognitive Battery); **MEB** (Mary/Eddie/Bill); **METT** (MicroExpression Training Tool); **MFT** (Multifamily Treatment); **MSCT** (Metacognitive and Social Cognition Training); **PE** (Psychoeducational); **PTP** (Psychoeducational Treatment Programme); **SCET** (Social Cognition Enhancement Training); **SCIT** (Social Cognitive and Interaction Training); **SCST** (Social Cognitive Skills Training); **SSD** (Schizophrenia Spectrum Disorders); **SCTP** (Social Cognition Training Program); **SoCog-MSRT** (Mental-State Reasoning Training for Social Cognitive Impairment); **SCT** (Social Cognitive Training); **TAR** (Training of Affect Recognition); **TAU** (Treatment As Usual); **tDCS** (transcranial Direct Current Stimulation); **ToM** (Theory of Mind); **TOMI** (Theory of Mind Intervention).

**eTable 4. Psychosocial interventions - Physical Exercise and Lifestyle Interventions**

| **Authors** | **Type of Document** | **Types Included of Studies** | **Level of evidence** | **Number of Included Studies**  **(Number of participants and diagnoses)** | **Intervention** | **Main Findings** |
| --- | --- | --- | --- | --- | --- | --- |
| Vancampfort D et al., 2012. [94] | Systematic Review | RCTs | I | 10  (322 SSD) | Exercise interventions | According to this systematic review, there is evidence that aerobic exercise improves short-term memory. |
| Firth J et al., 2015. [95] | Meta-analysis | Meta-analysis: RCTs | I | 17  (659 non-affective psychosis or FEP) | Exercise interventions | Symptoms were significantly reduced by interventions using around 90 min of moderate-to-vigorous exercise per week (SMD: 0.72, 95% CI −1.14 to −0.29). In one study it was observed that exercise improves verbal short-term memory by 34% (p < 0.05). |
| Dauwan M et al., 2016.  [96] | Meta-analysis | Controlled and non-controlled trials | I | 29  (1109 SSD) | Exercise interventions | Yoga specifically improved the cognitive subdomain “long-term memory” (k = 2, n = 184: Hedges’ g = .32, P < .05), whereas exercise in general or in any other form had no effect on cognition. |
| Firth J et al., 2017. [97] | Systematic Review | Independent trials | I | 14  (423 non-affective psychosis or FEP) | Exercise interventions | The cognitive benefits of exercise in schizophrenia may be due to exercise stimulating neurogenesis, perhaps by up-regulating BDNF, although current evidence is insufficient to draw definitive conclusions. |
| Firth J et al., 2017.  [98] | Meta-analysis | RCTs | I | 10  (385 SSD) | Exercise interventions | Exercise significantly improved global cognition (g = 0.33, 95% CI = 0.13–0.53, P = .001) with no statistical heterogeneity (I2 = 0%). Meta-regression analyses indicated that greater amounts of exercise are associated with larger improvements in global cognition (β = .005, P = .065). Interventions which were supervised by physical activity professionals were also more effective (g = 0.47, P < .001). In particular, exercise significantly improved the cognitive domains of working memory (g = 0.39, P = .024, N = 7, n = 282), social cognition (g = 0.71, P = .002, N = 3, n = 81), and attention/vigilance (g = 0.66, P = .005, N = 3, n = 104). Effects on processing speed, verbal memory, visual memory and reasoning and problem solving were not significant. |
| Li J et al., 2018. [99] | Meta-analysis | RCTs | I | 7  (679 SSD) | Mindful exercise | Mindful exercise was more beneficial over non-mindful exercise on some outcomes of symptoms and cognitive performance for schizophrenia. There were significant differences in favour of mindful exercise in "working memory" (1 RCT, n = 194, MD 0.39, low-quality). For outcomes of "attention" and social functioning, there was no clear difference. |
| Van der Stouwe ECD et al., 2018. [100] | Systematic review | RCTs | I | 15  (369 SSD and HC) | Exercise interventions | Comparing individuals with a schizophrenia spectrum disorder and healthy individuals within a similar age range, most studies found similar effects of exercise on hippocampal volume and white matter tracts for both groups, although the effect in schizophrenia spectrum disorders may be attenuated which is in line with previous literature on brain plasticity. The current review indicates a lack of studies investigating neural correlates other than the hippocampus. |
| Aucoin M et al., 2020. [101] | Systematic review | 25 clinical trials + 4 protocols | I | 29  (4448 psychotic disorders) | Diet modifications | Further research assessing effectiveness and efficacy of clearly reported dietary interventions is warranted. For what concerns cognition in the present research 4 studies were identified: of these, half measured an improvement with dietary intervention whereas the other half describes no improvement not worsening. |
| Dauwan M et al., 2021.  [102] | Meta-analysis | RCTs | I | 122  (7231 AS, HD, idiopathic MS, PD, SCZ, UD) | Exercise interventions | Exercise is an efficacious and safe add-on therapeutic intervention showing a medium-sized effect on QoL and a large effect on mood in patients with chronic brain disorders, with a positive dose-response correlation. Exercise also improved several cognitive domains with small but significant effects. Exercise was superior to treatment as usual in improving QoL (k = 64, n = 4334, ES = 0.40, p < 0.0001), depressive symptoms (k = 60, n = 2909, ES = 0.78, p < 0.0001), the cognitive domains attention and working memory (k = 21, n = 1313, ES = 0.24, p < 0.009), executive functioning (k = 14, n = 977, ES = 0.15, p = 0.013), memory (k = 12, n = 994, ES = 0.12, p = 0.038) and psychomotor speed (k = 16, n = 896, ES = 0.23, p = 0.003). Meta-regression showed a dose–response effect for exercise time (min/week) on depressive symptoms (β = 0.007, p = 0.012). 69% of the studies that reported on safety, found no complications. |
| Fernández-Abascal B et al., 2021. [103] | Meta-analysis | RCTs | I | 53  (SSD or FEP) | Exercise interventions and diet interventions and psychotherapy | Post-intervention benefit was found for many cognitive domains: for example, WAIS (Wechsler Adult Intelligence Scale) forward and backward digit span tests. Significant effect was observed at the end of intervention, of small size for digit span forward (g = 0.309, 95% CI = 0.062 to 0.556, p = 0.014) and medium size for the backward modality (g = 0.621, 95% CI = 0.288 to 0.953, p < 0.001). |
| Millman LSM et al., 2021.  [104] | Systematic review | RCTs | I | 15  (158 depression, 457 SCZ, 221 autism, 24 somatoform disorder) | DMT | A total of 5 studies included participants diagnosed with schizophrenia, and 2 studies reported positive effects on cognitive outcomes. |

**AD** (Alzheimer’s Disease); **BDNF** (Brain Derived Neurotropic Factor); **CI** (Confidence Interval); **DMT** (Dance Movement Therapy); **ES** (Effect Size); **FEP** (First Episode Psychoses); **HC** (Healthy Controls); **HD** (Huntington’s Disease); **MD** (Mean Difference); **MS** (Multiple Sclerosis); **PD** (Parkinson’s Disease); **QoL** (Quality of Life); **RCTs** (Randomized Controlled Trials); **SCZ** (Schizophrenia); **SMD** (Standardized Mean Difference); **SSD** (Schizophrenia Spectrum Disorders); **UD** (Unipolar Depression).

**eTable 5. Psychosocial interventions - Other Psychosocial Interventions**

| **Authors** | **Type of Document** | **Types of Included Studies** | **Level of evidence** | **Number of Included Studies**  **(Number of participants and diagnoses)** | **Intervention** | **Main Findings** |
| --- | --- | --- | --- | --- | --- | --- |
| Mössler, K et al., 2011. [105] | Meta-Analsysis | RCTs | I | 8  (483 SSD) | Music Therapy | Results suggest that music therapy could have a limited positive effect on cognitive performance on the basis of 1 study (1 RCT, n = 67). A positive effect of music therapy was observed in the attention domain measured with PASAT (SMD = 0.72, 95% CI = 0.22 to 1.21, p = 0.005). CCPT scores measuring vigilance and attention did not show a significant effect (SMD = 0.25, 95% CI = ‐0.23 to 0.74). Similarly, no significant effects could be found for memory measured with the WMS-R (SMD = 0.43, 95% CI = ‐0.06 to 0.92) and abstract thinking measured with BCST (SMD = 0.09, 95% CI = ‐0.39 to 0.58). |
| Geretsegger, M et al., 2017.  [106] | Meta-analysis | RCTs | I | 18  (1215 SSD) | Music Therapy+ standard care | A positive effect of music therapy was observed in the attention domain measured with PASAT on the basis of one study (1 RCT, n = 67, SMD = 0.72, 95% CI = 0.22 to 1.21, p = 0.005). CCPT scores measuring vigilance and attention did not show a significant effect (SMD 0.25, 95% CI = ‐0.23 to 0.74). Similarly, no significant effects could be found for memory measured with the WMS-R (SMD 0.43, 95% CI ‐0.06 to 0.92) and abstract thinking measured with BCST (SMD 0.09, 95% CI ‐0.39 to 0.58).  On the basis of one study a positive effect of music therapy was observed on memory (1 RCT, n = 60, SMD 0.58, 95% CI = 0.06 to 1.09).  No significant effect short-term effect was observed for abstract thinking measured with WCST‐Cc (2 RCTs, n = 90, SMD = ‐0.02, 95% CI = ‐0.07 to 0.03), but a significant positive effects was observed at medium term (1 RCT, n = 30, SMD = 1.18, 95% CI = 0.33 to 2.03). |
| Potes, A et al., 2018. [107] | Systematic review | 6 RCTs + 7 prospective studies + 1 retrospective study | I | 14  (Psychotic disorders) | Mindfulness | One small prospective study (n = 10) measured cognitive performance with the MCCB and reported a significant positive effect of a mindfulness intervention in working memory with a large effect size (d = 0.84). |
| Rus-Calafell M et al., 2018. [108] | Systematic review | Clinical trials | I | 50  (Psychoses) | VR | VR is a promising method to be used in the assessment of neurocognitive deficits and the study of relevant clinical symptoms. Furthermore, preliminary findings suggest that it can be applied to the delivery of cognitive rehabilitation, social skills training interventions and virtual reality-assisted therapies for psychosis. |
| van den Noort M et al., 2018. [109] | Systematic Review | Clinical trials | I | 26  (1181 SCZ) | Acupuncture | Most studies showed limited evidence for the use of acupuncture as add-on therapy in the treatment of the positive, negative, and cognitive symptoms, but beneficial effects have been reported in the treatment of the accompanying sleep disorders. |
| Roberts MT et al., 2021. [110] | Systematic review | RCTs | I | 7  (468 SSD) | Video games | Non-exergames may have a less beneficial effect on cognitive functioning than cognitive remediation, but they have comparable effects for all other outcomes. |

**BCST** (Berg’s Card Sorting Test); BDZ (Benzodiazepines); **CCPT** (Conners Continuous Performance Task); **CI** (Confidence Interval); **MCCB** (MATRICS Consensus Cognitive Battery); **PASAT** (Paced Auditory Serial Addition Task); **RCTs** (Randomized Controlled Trials); **SMD** (Standardized Mean Difference); **VR** (Virtual Reality); **WMS-R** (Wechsler Memory Scale-Revised).

**eTable 6. Somatic interventions- Non-invasive Brain Stimulation Techniques**

| **Authors** | **Type of Document** | **Types of Included Studies** | **Level of evidence** | **Number of Included Studies**  **(Number of participants and diagnoses)** | **Intervention** | **Main Findings** |
| --- | --- | --- | --- | --- | --- | --- |
| Brunoni AR et al., 2014. [111] | Meta-analysis | RCTs | I | 12  (805 HC, PD, MDD, SCZ) | rTMS and tDCS to increase DLPFC activity | rTMS of the DLPFC significantly improved all measures of WM performance whereas tDCS significantly improved RT, but not the percentage of correct and error responses. Mechanistic insights on the role of DLPFC in WM are further discussed, as well as how NIBS techniques could be used in neuropsychiatric samples presenting WM deficits, such as major depression, dementia and schizophrenia. |
| Mondino M et al., 2014. [112] | Systematic review | Case reports and RCTs | I | 9 on SCZ  (83 SCZ) | tDCS | Results of this systematic review report that tDCS could modulate cognitive deficits and symptoms in patients by acting on specific brain areas or networks. |
| Dougall N et al., 2015. [113] | Systematic review | RCTs | I | 41  (1473 SCZ) | TMS | According to the results of this systematic review, prefrontal TBS TMS does not appear to be superior to sham TMS in improving global state and cognitive state in schizophrenia. |
| Wang W et al., 2015. [114] | Meta-analysis | RCTs | I | 18  (1394 treatment-resistant SCZ) | Antipsychotic medications + ECT | Five studies reported results of cognitive functioning: two studies used the Mini-Mental State Examination one study used the Wisconsin Card Sorting Test and two studies used the Wechsler Memory Scale. Four studies were pooled for quantitative synthesis, with meta-analysis reporting no significant difference between treatment and control (SMD -0.28, 95% CI= -0.77 to 0.20, p = 0.25). |
| Hasan A et al., 2016. [115] | Systematic Review | 27 double-blind  controlled trials and 6 open-label trials | I | 33  (1084 SCZ) | Repetitive NIBS (rTMS and tDCS) | A beneficial effect of frontal NIBS could not be clearly established. No evidence for a cognitive disruptive effect of NIBS (temporal lobe) in schizophrenia could be detected. |
| Kedzior KK et al., 2016. [116] | Systematic review | Open-label study; Randomised study; Single-blind, placebo controlled, crossover study; Case study; Double-blind, randomised, | I | 13  (241 HC, MDD, SCZ) | DTMS intervention | Compared to baseline, executive functions, visuospatial memory, and sustained attention improved after 20 sessions of DTMS in one open-label study with 15 schizophrenia patients receiving concurrent antipsychotics. Similarly, executive functioning also improved after 20 sessions of DTMS in one sham-controlled RCT. However, this cognitive improvement was not related to DTMS alone because it was observed in the active DTMS and sham groups. |
| Martin DM et al., 2016. [117] | Meta-analysis | RCTs | I | 30 – 8 on SCZ  (321 SCZ) | rTMS administered to the DLPFC | Active rTMS treatment was not associated with generalised gains across the majority of domains of cognitive functioning examined (Global cognitive function, executive function, attention, working memory, processing speed, visual memory, verbal memory and visuospatial ability).  Secondary analyses revealed a moderate sized positive effect for improved working memory in a small number of studies in patients with schizophrenia (k = 3, g = 0.507, 95 % CI = 0.183 to 0.831, p < .01). |
| Zheng W et al., 2016. [118] | Meta-analysis | RCTs | I | 11  (818 SCZ) | ECT combined with non-clozapine antipsychotic medications | Adjunctive ECT was superior to antipsychotic monotherapy regarding symptomatic improvement at last-observation endpoint with an SMD of -0.67 (p<0.00001; I2 = 62%). However, the ECT-antipsychotic combination caused more headache (p = 0.02) with an NNH of 6 (CI = 4–11) and memory impairment (p = 0.001) with an NNH of 3 (CI = 2–5). |
| Ali SA et al., 2019. [119] | Systematic review | RCTs | I | 28  (SCZ) | ECT | Studies considered in this review suggest that while ECT in schizophrenia is a safe treatment modality, the potential for cognitive impairment must always be carefully weighed. |
| Hauer L et al., 2019 [120] | Systematic review | Clinical trials | I | 21  (318 MDD, 129 SCZ, 35 ADHD, 52 AD, 170 autism, 57 addiction) | rTMS | No evidence for cognitive adverse effects was found in all the included rTMS studies. Several studies showed a significant improvement of attentional function in patients with depression and schizophrenia. |
| Iimori T et al., 2019. [121] | Systematic Review | RCTs | I | 11  (594 MDD, SCZ, AD) | rTMS | This review demonstrated that prefrontal rTMS could exert pro-cognitive effects on executive function and attention in some patients with depression but inconsistent cognitive impacts in any of the examined domains especially in patients with schizophrenia and Alzheimer's disease. |
| Jiang Y et al., 2019. [122] | Meta-analysis | Clinical Trials | I | 9  (351 SCZ) | rTMS | A significant efficacy of high-frequency rTMS on working memory in SCZs was found compared to sham stimulation (SMD = 0.34, p = 0.009). Specifically, rTMS treatment positioned on the left DLPFC, with a total pluses <30,000 was significantly more effective in improving the working memory (SMD = 0.33, p = 0.03). No improvement was found in other cognitive domains such as executive function, attention, processing speed, and language function. Additionally, the result showed that rTMS stimulation improved executive function at a trend level compared with the sham stimulation (SMD = 0.23, 95% CI, −0.03–0.49, P = 0.08).  For the follow-up observations, high-frequency rTMS had long-lasting sustained effects on working memory (SMD = 0.45, p = 0.01) and language function (SMD = 0.77, p = 0.02) in SCZ. |
| Begemann MJ et al., 2020. [123] | Meta-analysis | RCTs | I | 82  (2784 dementia, MDD, SCZ, MS, PD, stroke, TBI) | TMS / tDCS | For working memory, both TMS (ES = 0.17, p = 0.015) and tDCS (ES = 0.17, p = 0.021) showed small but significant effects. Age positively moderated the effect of TMS. tDCS was superior to sham for attention/vigilance (ES = 0.20, p = 0.020). These significant effects did not differ across the type of brain disorder (schizophrenia, depression, dementia, Parkinson's disease, stroke, traumatic brain injury, and multiple sclerosis). Results were not significant for the other five cognitive domains. |
| Kostova R., 2020. [124] | Systematic Review | RCTs (29) and Non-controlled Clinical Trials (3) | I | 32  (842 SSD) | tDCS | Most studies (n = 21) identified significant behavioural and neural effects on a range of cognitive functions including working memory, attention and social cognition. However, no tDCS parameters (electrode montage, stimulation protocol, type and intensity) that clearly mediated effects on cognitive deficits were found. |
| Narita Z et al., 2020. [125] | Meta-Analysis | RCTs | I | 9  (270 SSD) | tDCS | This meta-analysis demonstrated a significant mean effect of tDCS on working memory (SMD = 0.49, 95% CI = 0.16 to 0.83), while non-significant results were produced for other domains. |
| Yu L et al., 2020. [126] | Meta-analysis | RCTs | I | 7  (256 SSD) | tDCS | This study reported a trend indicating that active tDCS might improve cognitive impairment (SMD -0.21, 95% CI = -0.46 to 0.04), but the overall meta-analysis failed to obtain statistically significant results. |
| Ciullo V et al., 2021. [127] | Systematic review | Clinical trials | I | 41  (AD, MDD, BD, SSD) | tDCS | tDCS has the capacity to enhance processing speed, working memory, and executive functions in patients with mood and schizophrenia-spectrum disorders. |
| Laskov O et al., 2021. [128] | Systematic review | 11 open-label or single-blind studies  and 12 double-blind controlled studies | I | 23  (1001 MDD, autism, BD, SCZ, OCD, ADHD, CNP) | dTMS | Improvement in executive functions and sustained attention (frontal  and frontoparietal-related tasks) was observed after 20 dTMS sessions in one open-label study with 15 schizophrenia patients. Another  sham-controlled study showed an improvement in executive  functions, but there was no difference between active and sham  groups, and therefore cognitive changes could not be related to dTMS  application alone. |
| Liu Y et al., 2021. [129] | Meta-analysis | RCTs | I | 12  (429 SSD) | tES | This meta-analysis demonstrated a significant efficacy of tES compared to sham tES in schizophrenia on working memory in follow up (SMD 0.33, 95% CI = 0.04 to 0.62, p = 0.02; n = 190, 4 studies; I 2 = 33%), while non-significant results were observed for working memory assessed immediately after the last tES session (SMD 0.14, 95% CI = -0.12 to 0.41; p = 0.30, n = 417, 11 studies; I2 = 41%). |
| Sloan NP et al., 2021. [130] | Meta-Analysis | RCTs | I | 22  (708 SSD) | rTMS and tES | This meta-analysis showed that compared to sham/placebo stimulation, neither TMS nor tES significantly improved working memory. This was found when working memory was measured with respect to the accuracy on working memory tasks (TMS studies: Hedges’ g = 0.112, CI95: −0.082, 0.305, p = .257; tES studies Hedges’ g = 0.080, CI95: −0.117, 0.277, p = .427) or the speed working memory tasks were completed (rTMS studies: Hedges’ g = 0.233, CI95: −0.212, 0.678, p = .305; tES studies Hedges’ g = −0.016, CI95: −0.204, 0.173, p = .871). For tES studies, meta-regression analysis found that studies with a larger number of stimulation sessions were associated with larger treatment effects; this association was not found for TMS studies. |
| Sun CH et al., 2021. [131] | Meta-analysis | RCT | I | 12  (418 SSD) | tDCS | Adjunctive tDCS outperformed the comparator in improving working memory deficits (12 RCTs, n = 383, SMD=0.34, 95% CI: 0.03, 0.65;  I 2 = 52%, p = 0.03), but not in MCCB total scores (SMD = 0.29,  95% CI: − 0.08, 0.66; I 2 = 20%, p = 0.13). However, the superiority of adjunctive tDCS disappeared after excluding one RCT with outlying effect size (SMD = 0.26, 95% CI: − 0.04, 0.56; I 2 = 43%, p = 0.09). No significant differences were found with regard to the other 6 MCCB cognitive domains including speed of processing, attention/vigilance, verbal learning and memory, visual learning and memory, reasoning and problem solving, and social cognition between adjunctive tDCS and the control groups (SMD = 0.02–0.20; p = 0.20–0.89) |
| Yamada Y et al., 2021. [132] | Systematic review | RCTs | I | 5  (244 SCZ and MDD) | tDCS and rTMS | tDCS and rTMS may enhance some domains of social cognition in patients with psychiatric disorders. Specifically, 3 RCTs showed a significant effect of tDCS and rTMS targeting the left DLPFC on the emotion recognition domain in patients with schizophrenia. In addition, rTMS on the right IPL ameliorated social perception impairments of schizophrenia. |

**AD** (Alzheimer’s Disease); **ADHD** (Attention Deficit Hyperactivity Disorder); **BD** (Bipolar Disorder); **CI** (Confidence Interval); **CNP** (Central Neuropathic Pain); **DLPFC** (Dorso-Lateral Pre-Frontal Cortex); **DTMS** (Deep Transcranial Magnetic Stimulation); **ECT** (Electroconvulsive Therapy); **ES** (Effect Size); **HC** (Healthy Controls); **IPL** (Inferior Parietal Lobe); **OCD** (Obsessive Compulsive Disorder); **MCCB** (MATRICS Consensus Cognitive Battery); **MDD** (Major Depressive Disorder); **MMSE** (Mini Mental Status Examination); **NIBS** (Non Invasive Brain Stimulation); **NNH** (Number Needed to Harm); **NNT** (Number Needed to Treat); **MS** (Multiple Sclerosis); **PANSS** (Positive And Negative Symptoms Scale); **PD** (Parkinson’s Disease); **RCTs** (Randomized Controlled Trials), **RR** (Relative Risk); **RT** (Response Times); **rTMS** (repetitive Transcranial Magnetic Stimulation); **SMD** (Standardized Mean Difference); **SCZ** (Schizophrenia); **SSD** (Schizophrenia Spectrum Disorder); **TBS** (Theta Burst Stimulation); **TBI** (Traumatic Brain Injury); **tDCS** (transcranial Direct Current Stimulation); **tES** (transcranial Electrical Stimulation); **TMS** (Transcranial Magnetic Stimulation); **WCST** (Wisconsin Card Sorting Test); **WMS-R** (Wechsler Memory Scale-Revised); **WMD** (Weighted Mean Difference).

[1] Keefe RS, Silva SG, Perkins DO, Lieberman JA. The effects of atypical antipsychotic drugs on neurocognitive impairment in schizophrenia: a review and meta-analysis. Schizophr Bull 1999;25:201–22. https://doi.org/10.1093/oxfordjournals.schbul.a033374.

[2] Kennedy E, Song F, Hunter R, Clarke A, Gilbody S. Risperidone versus typical antipsychotic medication for schizophrenia. Cochrane Database Syst Rev 2000:CD000440. https://doi.org/10.1002/14651858.CD000440.

[3] Mishara AL, Goldberg TE. A meta-analysis and critical review of the effects of conventional neuroleptic treatment on cognition in schizophrenia: opening a closed book. Biol Psychiatry 2004;55:1013–22. https://doi.org/10.1016/j.biopsych.2004.01.027.

[4] Woodward ND, Purdon SE, Meltzer HY, Zald DH. A meta-analysis of neuropsychological change to clozapine, olanzapine, quetiapine, and risperidone in schizophrenia. Int J Neuropsychopharmacol 2005;8:457–72. https://doi.org/10.1017/S146114570500516X.

[5] Woodward ND, Purdon SE, Meltzer HY, Zald DH. A meta-analysis of cognitive change with haloperidol in clinical trials of atypical antipsychotics: dose effects and comparison to practice effects. Schizophr Res 2007;89:211–24. https://doi.org/10.1016/j.schres.2006.08.021.

[6] Riedel M, Schennach-Wolff R, Musil R, Dehning S, Cerovecki A, Opgen-Rhein M, et al. Neurocognition and its influencing factors in the treatment of schizophrenia-effects of aripiprazole, olanzapine, quetiapine and risperidone. Hum Psychopharmacol 2010;25:116–25. https://doi.org/10.1002/hup.1101.

[7] Barry SJ, Gaughan TM, Hunter R. Schizophrenia. BMJ Clin Evid 2012;2012:1007.

[8] Désaméricq G, Schurhoff F, Meary A, Szöke A, Macquin-Mavier I, Bachoud-Lévi AC, et al. Long-term neurocognitive effects of antipsychotics in schizophrenia: a network meta-analysis. Eur J Clin Pharmacol 2014;70:127–34. https://doi.org/10.1007/s00228-013-1600-y.

[9] Gabay AS, Kempton MJ, Mehta MA. Facial affect processing deficits in schizophrenia: a meta-analysis of antipsychotic treatment effects. J Psychopharmacol Oxf Engl 2015;29:224–9. https://doi.org/10.1177/0269881114560184.

[10] Nielsen RE, Levander S, Kjaersdam Telléus G, Jensen SOW, Østergaard Christensen T, Leucht S. Second-generation antipsychotic effect on cognition in patients with schizophrenia--a meta-analysis of randomized clinical trials. Acta Psychiatr Scand 2015;131:185–96. https://doi.org/10.1111/acps.12374.

[11] Schoretsanitis G, Kane JM, Ruan C-J, Spina E, Hiemke C, de Leon J. A comprehensive review of the clinical utility of and a combined analysis of the clozapine/norclozapine ratio in therapeutic drug monitoring for adult patients. Expert Rev Clin Pharmacol 2019;12:603–21. https://doi.org/10.1080/17512433.2019.1617695.

[12] Ohi K, Muto Y, Sugiyama S, Shioiri T. Safety and Efficacy in Randomized Controlled Trials of Second-Generation Antipsychotics Versus Placebo for Cognitive Impairments in Schizophrenia: A Meta-Analysis. J Clin Psychopharmacol 2022;42:227–9. https://doi.org/10.1097/JCP.0000000000001232.

[13] Baldez DP, Biazus TB, Rabelo-da-Ponte FD, Nogaro GP, Martins DS, Kunz M, et al. The effect of antipsychotics on the cognitive performance of individuals with psychotic disorders: Network meta-analyses of randomized controlled trials. Neurosci Biobehav Rev 2021;126:265–75. https://doi.org/10.1016/j.neubiorev.2021.03.028.

[14] Haime Z, Watson AJ, Crellin N, Marston L, Joyce E, Moncrieff J. A systematic review of the effects of psychiatric medications on social cognition. BMC Psychiatry 2021;21:597. https://doi.org/10.1186/s12888-021-03545-z.

[15] Kishimoto T, Hagi K, Kurokawa S, Kane JM, Correll CU. Long-acting injectable versus oral antipsychotics for the maintenance treatment of schizophrenia: a systematic review and comparative meta-analysis of randomised, cohort, and pre-post studies. Lancet Psychiatry 2021;8:387–404. https://doi.org/10.1016/S2215-0366(21)00039-0.

[16] Chua WL, de Izquierdo SA, Kulkarni J, Mortimer A. Estrogen for schizophrenia. Cochrane Database Syst Rev 2005:CD004719. https://doi.org/10.1002/14651858.CD004719.pub2.

[17] Tuominen HJ, Tiihonen J, Wahlbeck K. Glutamatergic drugs for schizophrenia: a systematic review and meta-analysis. Schizophr Res 2005;72:225–34. https://doi.org/10.1016/j.schres.2004.05.005.

[18] Ferreri F, Agbokou C, Gauthier S. Cognitive dysfunctions in schizophrenia: potential benefits of cholinesterase inhibitor adjunctive therapy. J Psychiatry Neurosci JPN 2006;31:369–76.

[19] Premkumar TS, Pick J. Lamotrigine for schizophrenia. Cochrane Database Syst Rev 2006:CD005962. https://doi.org/10.1002/14651858.CD005962.pub2.

[20] Tuominen HJ, Tiihonen J, Wahlbeck K. Glutamatergic drugs for schizophrenia. Cochrane Database Syst Rev 2006:CD003730. https://doi.org/10.1002/14651858.CD003730.pub2.

[21] Chouinard S, Sepehry AA, Stip E. Oral cholinesterase inhibitor add-on therapy for cognitive enhancement in schizophrenia: a quantitative systematic review, Part I. Clin Neuropharmacol 2007;30:169–82. https://doi.org/10.1097/WNF.0b013e31802fa61a.

[22] Stip E, Sepehry AA, Chouinard S. Add-on therapy with acetylcholinesterase inhibitors for memory dysfunction in schizophrenia: a systematic quantitative review, part 2. Clin Neuropharmacol 2007;30:218–29. https://doi.org/10.1097/WNF.0b013e318059be76.

[23] Saavedra-Velez C, Yusim A, Anbarasan D, Lindenmayer J-P. Modafinil as an adjunctive treatment of sedation, negative symptoms, and cognition in schizophrenia: a critical review. J Clin Psychiatry 2009;70:104–12. https://doi.org/10.4088/jcp.07r03982.

[24] Ribeiz SRI, Bassitt DP, Arrais JA, Avila R, Steffens DC, Bottino CMC. Cholinesterase inhibitors as adjunctive therapy in patients with schizophrenia and schizoaffective disorder: a review and meta-analysis of the literature. CNS Drugs 2010;24:303–17. https://doi.org/10.2165/11530260-000000000-00000.

[25] Singh J, Kour K, Jayaram MB. Acetylcholinesterase inhibitors for schizophrenia. Cochrane Database Syst Rev 2012;1:CD007967. https://doi.org/10.1002/14651858.CD007967.pub2.

[26] Choi K-H, Wykes T, Kurtz MM. Adjunctive pharmacotherapy for cognitive deficits in schizophrenia: meta-analytical investigation of efficacy. Br J Psychiatry J Ment Sci 2013;203:172–8. https://doi.org/10.1192/bjp.bp.111.107359.

[27] Kishi T, Iwata N. NMDA receptor antagonists interventions in schizophrenia: Meta-analysis of randomized, placebo-controlled trials. J Psychiatr Res 2013;47:1143–9. https://doi.org/10.1016/j.jpsychires.2013.04.013.

[28] Scoriels L, Jones PB, Sahakian BJ. Modafinil effects on cognition and emotion in schizophrenia and its neurochemical modulation in the brain. Neuropharmacology 2013;64:168–84. https://doi.org/10.1016/j.neuropharm.2012.07.011.

[29] Vernon JA, Grudnikoff E, Seidman AJ, Frazier TW, Vemulapalli MS, Pareek P, et al. Antidepressants for cognitive impairment in schizophrenia--a systematic review and meta-analysis. Schizophr Res 2014;159:385–94. https://doi.org/10.1016/j.schres.2014.08.015.

[30] Andrade C, Kisely S, Monteiro I, Rao S. Antipsychotic augmentation with modafinil or armodafinil for negative symptoms of schizophrenia: systematic review and meta-analysis of randomized controlled trials. J Psychiatr Res 2015;60:14–21. https://doi.org/10.1016/j.jpsychires.2014.09.013.

[31] Heringa SM, Begemann MJH, Goverde AJ, Sommer IEC. Sex hormones and oxytocin augmentation strategies in schizophrenia: A quantitative review. Schizophr Res 2015;168:603–13. https://doi.org/10.1016/j.schres.2015.04.002.

[32] Iseger TA, Bossong MG. A systematic review of the antipsychotic properties of cannabidiol in humans. Schizophr Res 2015;162:153–61. https://doi.org/10.1016/j.schres.2015.01.033.

[33] Iwata Y, Nakajima S, Suzuki T, Keefe RSE, Plitman E, Chung JK, et al. Effects of glutamate positive modulators on cognitive deficits in schizophrenia: a systematic review and meta-analysis of double-blind randomized controlled trials. Mol Psychiatry 2015;20:1151–60. https://doi.org/10.1038/mp.2015.68.

[34] Rowe AR, Mercer L, Casetti V, Sendt K-V, Giaroli G, Shergill SS, et al. Dementia praecox redux: a systematic review of the nicotinic receptor as a target for cognitive symptoms of schizophrenia. J Psychopharmacol Oxf Engl 2015;29:197–211. https://doi.org/10.1177/0269881114564096.

[35] Terevnikov V, Joffe G, Stenberg J-H. Randomized Controlled Trials of Add-On Antidepressants in Schizophrenia. Int J Neuropsychopharmacol 2015;18:pyv049. https://doi.org/10.1093/ijnp/pyv049.

[36] Magalhães PVS, Dean O, Andreazza AC, Berk M, Kapczinski F. Antioxidant treatments for schizophrenia. Cochrane Database Syst Rev 2016;2:CD008919. https://doi.org/10.1002/14651858.CD008919.pub2.

[37] Zheng W, Xiang Y-Q, Li X-B, Ungvari GS, Chiu HFK, Sun F, et al. Adjunctive huperzine A for cognitive deficits in schizophrenia: a systematic review and meta-analysis. Hum Psychopharmacol 2016;31:286–95. https://doi.org/10.1002/hup.2537.

[38] Bürkner P-C, Williams DR, Simmons TC, Woolley JD. Intranasal Oxytocin May Improve High-Level Social Cognition in Schizophrenia, But Not Social Cognition or Neurocognition in General: A Multilevel Bayesian Meta-analysis. Schizophr Bull 2017;43:1291–303. https://doi.org/10.1093/schbul/sbx053.

[39] Correll CU, Rubio JM, Inczedy-Farkas G, Birnbaum ML, Kane JM, Leucht S. Efficacy of 42 Pharmacologic Cotreatment Strategies Added to Antipsychotic Monotherapy in Schizophrenia: Systematic Overview and Quality Appraisal of the Meta-analytic Evidence. JAMA Psychiatry 2017;74:675–84. https://doi.org/10.1001/jamapsychiatry.2017.0624.

[40] Di Iorio G, Baroni G, Lorusso M, Montemitro C, Spano MC, di Giannantonio M. Efficacy of Memantine in Schizophrenic Patients: A Systematic Review. J Amino Acids 2017;2017:7021071. https://doi.org/10.1155/2017/7021071.

[41] Jin Y, Wang Q, Wang Y, Liu M, Sun A, Geng Z, et al. Alpha7 nAChR Agonists for Cognitive Deficit and Negative Symptoms in Schizophrenia: A Meta-analysis of Randomized Double-blind Controlled Trials. Shanghai Arch Psychiatry 2017;29:191–9. https://doi.org/10.11919/j.issn.1002-0829.217044.

[42] Kishi T, Matsuda Y, Iwata N. Memantine add-on to antipsychotic treatment for residual negative and cognitive symptoms of schizophrenia: a meta-analysis. Psychopharmacology (Berl) 2017;234:2113–25. https://doi.org/10.1007/s00213-017-4616-7.

[43] Lewis AS, van Schalkwyk GI, Bloch MH. Alpha-7 nicotinic agonists for cognitive deficits in neuropsychiatric disorders: A translational meta-analysis of rodent and human studies. Prog Neuropsychopharmacol Biol Psychiatry 2017;75:45–53. https://doi.org/10.1016/j.pnpbp.2017.01.001.

[44] Solmi M, Veronese N, Thapa N, Facchini S, Stubbs B, Fornaro M, et al. Systematic review and meta-analysis of the efficacy and safety of minocycline in schizophrenia. CNS Spectr 2017;22:415–26. https://doi.org/10.1017/S1092852916000638.

[45] Kishi T, Ikuta T, Oya K, Matsunaga S, Matsuda Y, Iwata N. Anti-Dementia Drugs for Psychopathology and Cognitive Impairment in Schizophrenia: A Systematic Review and Meta-Analysis. Int J Neuropsychopharmacol 2018;21:748–57. https://doi.org/10.1093/ijnp/pyy045.

[46] Matthews PRL, Horder J, Pearce M. Selective noradrenaline reuptake inhibitors for schizophrenia. Cochrane Database Syst Rev 2018;1:CD010219. https://doi.org/10.1002/14651858.CD010219.pub2.

[47] Santos B, González-Fraile E, Zabala A, Guillén V, Rueda JR, Ballesteros J. Cognitive improvement of acetylcholinesterase inhibitors in schizophrenia. J Psychopharmacol Oxf Engl 2018;32:1155–66. https://doi.org/10.1177/0269881118805496.

[48] Sinkeviciute I, Begemann M, Prikken M, Oranje B, Johnsen E, Lei WU, et al. Efficacy of different types of cognitive enhancers for patients with schizophrenia: a meta-analysis. NPJ Schizophr 2018;4:22. https://doi.org/10.1038/s41537-018-0064-6.

[49] Soria V, González-Rodríguez A, Huerta-Ramos E, Usall J, Cobo J, Bioque M, et al. Targeting hypothalamic-pituitary-adrenal axis hormones and sex steroids for improving cognition in major mood disorders and schizophrenia: a systematic review and narrative synthesis. Psychoneuroendocrinology 2018;93:8–19. https://doi.org/10.1016/j.psyneuen.2018.04.012.

[50] Tan B-L, Lee S-A, Lee J. Social cognitive interventions for people with schizophrenia: A systematic review. Asian J Psychiatry 2018;35:115–31. https://doi.org/10.1016/j.ajp.2016.06.013.

[51] Zheng W, Li X-H, Cai D-B, Yang X-H, Ungvari GS, Ng CH, et al. Adjunctive azapirone for schizophrenia: A meta-analysis of randomized, double-blind, placebo-controlled trials. Eur Neuropsychopharmacol J Eur Coll Neuropsychopharmacol 2018;28:149–58. https://doi.org/10.1016/j.euroneuro.2017.11.007.

[52] Chang C-H, Lane H-Y, Tseng P-T, Chen S-J, Liu C-Y, Lin C-H. Effect of N-methyl-D-aspartate-receptor-enhancing agents on cognition in patients with schizophrenia: A systematic review and meta-analysis of double-blind randomised controlled trials. J Psychopharmacol Oxf Engl 2019;33:436–48. https://doi.org/10.1177/0269881118822157.

[53] Cho M, Lee TY, Kwak YB, Yoon YB, Kim M, Kwon JS. Adjunctive use of anti-inflammatory drugs for schizophrenia: A meta-analytic investigation of randomized controlled trials. Aust N Z J Psychiatry 2019;53:742–59. https://doi.org/10.1177/0004867419835028.

[54] Ortiz-Orendain J, Covarrubias-Castillo SA, Vazquez-Alvarez AO, Castiello-de Obeso S, Arias Quiñones GE, Seegers M, et al. Modafinil for people with schizophrenia or related disorders. Cochrane Database Syst Rev 2019;12:CD008661. https://doi.org/10.1002/14651858.CD008661.pub2.

[55] Solmi M, Fornaro M, Toyoshima K, Carvalho AF, Köhler CA, Veronese N, et al. Systematic review and exploratory meta-analysis of the efficacy, safety, and biological effects of psychostimulants and atomoxetine in patients with schizophrenia or schizoaffective disorder. CNS Spectr 2019;24:479–95. https://doi.org/10.1017/S1092852918001050.

[56] Zheng W, Cai D-B, Zhang Q-E, He J, Zhong L-Y, Sim K, et al. Adjunctive ondansetron for schizophrenia: A systematic review and meta-analysis of randomized controlled trials. J Psychiatr Res 2019;113:27–33. https://doi.org/10.1016/j.jpsychires.2019.02.024.

[57] Zheng W, Zhu X-M, Zhang Q-E, Cai D-B, Yang X-H, Zhou Y-L, et al. Adjunctive memantine for major mental disorders: A systematic review and meta-analysis of randomized double-blind controlled trials. Schizophr Res 2019;209:12–21. https://doi.org/10.1016/j.schres.2019.05.019.

[58] Chang C-H, Lin C-H, Liu C-Y, Chen S-J, Lane H-Y. Efficacy and cognitive effect of sarcosine (N-methylglycine) in patients with schizophrenia: A systematic review and meta-analysis of double-blind randomised controlled trials. J Psychopharmacol Oxf Engl 2020;34:495–505. https://doi.org/10.1177/0269881120908016.

[59] Dondé C, Brunelin J, Mondino M, Cellard C, Rolland B, Haesebaert F. The effects of acute nicotine administration on cognitive and early sensory processes in schizophrenia: a systematic review. Neurosci Biobehav Rev 2020;118:121–33. https://doi.org/10.1016/j.neubiorev.2020.07.035.

[60] Jeppesen R, Christensen RHB, Pedersen EMJ, Nordentoft M, Hjorthøj C, Köhler-Forsberg O, et al. Efficacy and safety of anti-inflammatory agents in treatment of psychotic disorders - A comprehensive systematic review and meta-analysis. Brain Behav Immun 2020;90:364–80. https://doi.org/10.1016/j.bbi.2020.08.028.

[61] Koola MM, Looney SW, Hong H, Pillai A, Hou W. Meta-analysis of randomized controlled trials of galantamine in schizophrenia: significant cognitive enhancement. Psychiatry Res 2020;291:113285. https://doi.org/10.1016/j.psychres.2020.113285.

[62] Kopelli E, Samara M, Siargkas A, Goulas A, Papazisis G, Chourdakis M. The role of cannabidiol oil in schizophrenia treatment. a systematic review and meta-analysis. Psychiatry Res 2020;291:113246. https://doi.org/10.1016/j.psychres.2020.113246.

[63] Tanzer T, Shah S, Benson C, De Monte V, Gore-Jones V, Rossell SL, et al. Varenicline for cognitive impairment in people with schizophrenia: systematic review and meta-analysis. Psychopharmacology (Berl) 2020;237:11–9. https://doi.org/10.1007/s00213-019-05396-9.

[64] Yolland CO, Hanratty D, Neill E, Rossell SL, Berk M, Dean OM, et al. Meta-analysis of randomised controlled trials with N-acetylcysteine in the treatment of schizophrenia. Aust N Z J Psychiatry 2020;54:453–66. https://doi.org/10.1177/0004867419893439.

[65] Zheng W, Xiang Y-Q, Cai D-B, Yang X-H, Zhang L, Zheng W, et al. Adjunctive Fluvoxamine for Schizophrenia: A Meta-analysis of Randomized Double-Blind, Placebo-Controlled Trials. J Clin Psychopharmacol 2020;40:386–90. https://doi.org/10.1097/JCP.0000000000001245.

[66] Ahmed S, Roth RM, Stanciu CN, Brunette MF. The Impact of THC and CBD in Schizophrenia: A Systematic Review. Front Psychiatry 2021;12:694394. https://doi.org/10.3389/fpsyt.2021.694394.

[67] Kuppili PP, Menon V, Sathyanarayanan G, Sarkar S, Andrade C. Efficacy of adjunctive D-Cycloserine for the treatment of schizophrenia: a systematic review and meta-analysis of randomized controlled trials. J Neural Transm Vienna Austria 1996 2021;128:253–62. https://doi.org/10.1007/s00702-020-02292-x.

[68] Recio-Barbero M, Segarra R, Zabala A, González-Fraile E, González-Pinto A, Ballesteros J. Cognitive Enhancers in Schizophrenia: A Systematic Review and Meta-Analysis of Alpha-7 Nicotinic Acetylcholine Receptor Agonists for Cognitive Deficits and Negative Symptoms. Front Psychiatry 2021;12:631589. https://doi.org/10.3389/fpsyt.2021.631589.

[69] Seetharam JC, Maiti R, Mishra A, Mishra BR. Efficacy and safety of add-on sodium benzoate, a D-amino acid oxidase inhibitor, in treatment of schizophrenia: A systematic review and meta-analysis. Asian J Psychiatry 2022;68:102947. https://doi.org/10.1016/j.ajp.2021.102947.

[70] Roder V, Mueller DR, Mueser KT, Brenner HD. Integrated psychological therapy (IPT) for schizophrenia: is it effective? Schizophr Bull 2006;32 Suppl 1:S81-93. https://doi.org/10.1093/schbul/sbl021.

[71] McGurk SR, Twamley EW, Sitzer DI, McHugo GJ, Mueser KT. A meta-analysis of cognitive remediation in schizophrenia. Am J Psychiatry 2007;164:1791–802. https://doi.org/10.1176/appi.ajp.2007.07060906.

[72] Roder V, Mueller DR, Schmidt SJ. Effectiveness of integrated psychological therapy (IPT) for schizophrenia patients: a research update. Schizophr Bull 2011;37 Suppl 2:S71-79. https://doi.org/10.1093/schbul/sbr072.

[73] Wykes T, Huddy V, Cellard C, McGurk SR, Czobor P. A meta-analysis of cognitive remediation for schizophrenia: methodology and effect sizes. Am J Psychiatry 2011;168:472–85. https://doi.org/10.1176/appi.ajp.2010.10060855.

[74] Kurtz MM, Richardson CL. Social cognitive training for schizophrenia: a meta-analytic investigation of controlled research. Schizophr Bull 2012;38:1092–104. https://doi.org/10.1093/schbul/sbr036.

[75] Kluwe-Schiavon B, Sanvicente-Vieira B, Kristensen CH, Grassi-Oliveira R. Executive functions rehabilitation for schizophrenia: a critical systematic review. J Psychiatr Res 2013;47:91–104. https://doi.org/10.1016/j.jpsychires.2012.10.001.

[76] Mueller DR, Schmidt SJ, Roder V. Integrated psychological therapy: effectiveness in schizophrenia inpatient settings related to patients’ age. Am J Geriatr Psychiatry Off J Am Assoc Geriatr Psychiatry 2013;21:231–41. https://doi.org/10.1016/j.jagp.2012.12.011.

[77] Paquin K, Wilson AL, Cellard C, Lecomte T, Potvin S. A systematic review on improving cognition in schizophrenia: which is the more commonly used type of training, practice or strategy learning? BMC Psychiatry 2014;14:139. https://doi.org/10.1186/1471-244X-14-139.

[78] Thorsen AL, Johansson K, Løberg E-M. Neurobiology of cognitive remediation therapy for schizophrenia: a systematic review. Front Psychiatry 2014;5:103. https://doi.org/10.3389/fpsyt.2014.00103.

[79] Revell ER, Neill JC, Harte M, Khan Z, Drake RJ. A systematic review and meta-analysis of cognitive remediation in early schizophrenia. Schizophr Res 2015;168:213–22. https://doi.org/10.1016/j.schres.2015.08.017.

[80] Isaac C, Januel D. Neural correlates of cognitive improvements following cognitive remediation in schizophrenia: a systematic review of randomized trials. Socioaffective Neurosci Psychol 2016;6:30054. https://doi.org/10.3402/snp.v6.30054.

[81] Wei Y-Y, Wang J-J, Yan C, Li Z-Q, Pan X, Cui Y, et al. Correlation Between Brain Activation Changes and Cognitive Improvement Following Cognitive Remediation Therapy in Schizophrenia: An Activation Likelihood Estimation Meta-analysis. Chin Med J (Engl) 2016;129:578–85. https://doi.org/10.4103/0366-6999.176983.

[82] Grant N, Lawrence M, Preti A, Wykes T, Cella M. Social cognition interventions for people with schizophrenia: a systematic review focussing on methodological quality and intervention modality. Clin Psychol Rev 2017;56:55–64. https://doi.org/10.1016/j.cpr.2017.06.001.

[83] Morin L, Franck N. Rehabilitation Interventions to Promote Recovery from Schizophrenia: A Systematic Review. Front Psychiatry 2017;8:100. https://doi.org/10.3389/fpsyt.2017.00100.

[84] Vass E, Fekete Z, Simon V, Simon L. Interventions for the treatment of theory of mind deficits in schizophrenia: Systematic literature review. Psychiatry Res 2018;267:37–47. https://doi.org/10.1016/j.psychres.2018.05.001.

[85] Kambeitz-Ilankovic L, Betz LT, Dominke C, Haas SS, Subramaniam K, Fisher M, et al. Multi-outcome meta-analysis (MOMA) of cognitive remediation in schizophrenia: Revisiting the relevance of human coaching and elucidating interplay between multiple outcomes. Neurosci Biobehav Rev 2019;107:828–45. https://doi.org/10.1016/j.neubiorev.2019.09.031.

[86] Prikken M, Konings MJ, Lei WU, Begemann MJH, Sommer IEC. The efficacy of computerized cognitive drill and practice training for patients with a schizophrenia-spectrum disorder: A meta-analysis. Schizophr Res 2019;204:368–74. https://doi.org/10.1016/j.schres.2018.07.034.

[87] Cella M, Price T, Corboy H, Onwumere J, Shergill S, Preti A. Cognitive remediation for inpatients with psychosis: a systematic review and meta-analysis. Psychol Med 2020;50:1062–76. https://doi.org/10.1017/S0033291720000872.

[88] Datta SS, Daruvala R, Kumar A. Psychological interventions for psychosis in adolescents. Cochrane Database Syst Rev 2020;7:CD009533. https://doi.org/10.1002/14651858.CD009533.pub2.

[89] Gandara V, Pineda JA, Shu I-W, Singh F. A Systematic Review of the Potential Use of Neurofeedback in Patients With Schizophrenia. Schizophr Bull Open 2020;1:sgaa005. https://doi.org/10.1093/schizbullopen/sgaa005.

[90] Jahn FS, Skovbye M, Obenhausen K, Jespersen AE, Miskowiak KW. Cognitive training with fully immersive virtual reality in patients with neurological and psychiatric disorders: A systematic review of randomized controlled trials. Psychiatry Res 2021;300:113928. https://doi.org/10.1016/j.psychres.2021.113928.

[91] Lejeune JA, Northrop A, Kurtz MM. A Meta-analysis of Cognitive Remediation for Schizophrenia: Efficacy and the Role of Participant and Treatment Factors. Schizophr Bull 2021;47:997–1006. https://doi.org/10.1093/schbul/sbab022.

[92] Vita A, Barlati S, Ceraso A, Nibbio G, Ariu C, Deste G, et al. Effectiveness, Core Elements, and Moderators of Response of Cognitive Remediation for Schizophrenia: A Systematic Review and Meta-analysis of Randomized Clinical Trials. JAMA Psychiatry 2021;78:848–58. https://doi.org/10.1001/jamapsychiatry.2021.0620.

[93] Yeo H, Yoon S, Lee J, Kurtz MM, Choi K. A meta-analysis of the effects of social-cognitive training in schizophrenia: The role of treatment characteristics and study quality. Br J Clin Psychol 2022;61:37–57. https://doi.org/10.1111/bjc.12320.

[94] Vancampfort D, Probst M, Helvik Skjaerven L, Catalán-Matamoros D, Lundvik-Gyllensten A, Gómez-Conesa A, et al. Systematic review of the benefits of physical therapy within a multidisciplinary care approach for people with schizophrenia. Phys Ther 2012;92:11–23. https://doi.org/10.2522/ptj.20110218.

[95] Firth J, Cotter J, Elliott R, French P, Yung AR. A systematic review and meta-analysis of exercise interventions in schizophrenia patients. Psychol Med 2015;45:1343–61. https://doi.org/10.1017/S0033291714003110.

[96] Dauwan M, Begemann MJH, Heringa SM, Sommer IE. Exercise Improves Clinical Symptoms, Quality of Life, Global Functioning, and Depression in Schizophrenia: A Systematic Review and Meta-analysis. Schizophr Bull 2016;42:588–99. https://doi.org/10.1093/schbul/sbv164.

[97] Firth J, Cotter J, Carney R, Yung AR. The pro-cognitive mechanisms of physical exercise in people with schizophrenia. Br J Pharmacol 2017;174:3161–72. https://doi.org/10.1111/bph.13772.

[98] Firth J, Stubbs B, Rosenbaum S, Vancampfort D, Malchow B, Schuch F, et al. Aerobic Exercise Improves Cognitive Functioning in People With Schizophrenia: A Systematic Review and Meta-Analysis. Schizophr Bull 2017;43:546–56. https://doi.org/10.1093/schbul/sbw115.

[99] Li J, Shen J, Wu G, Tan Y, Sun Y, Keller E, et al. Mindful exercise versus non-mindful exercise for schizophrenia: A systematic review and meta-analysis of randomized controlled trials. Complement Ther Clin Pract 2018;32:17–24. https://doi.org/10.1016/j.ctcp.2018.04.003.

[100] van der Stouwe ECD, van Busschbach JT, de Vries B, Cahn W, Aleman A, Pijnenborg GHM. Neural correlates of exercise training in individuals with schizophrenia and in healthy individuals: A systematic review. NeuroImage Clin 2018;19:287–301. https://doi.org/10.1016/j.nicl.2018.04.018.

[101] Aucoin M, LaChance L, Clouthier SN, Cooley K. Dietary modification in the treatment of schizophrenia spectrum disorders: A systematic review. World J Psychiatry 2020;10:187–201. https://doi.org/10.5498/wjp.v10.i8.187.

[102] Dauwan M, Begemann MJH, Slot MIE, Lee EHM, Scheltens P, Sommer IEC. Physical exercise improves quality of life, depressive symptoms, and cognition across chronic brain disorders: a transdiagnostic systematic review and meta-analysis of randomized controlled trials. J Neurol 2021;268:1222–46. https://doi.org/10.1007/s00415-019-09493-9.

[103] Fernández-Abascal B, Suárez-Pinilla P, Cobo-Corrales C, Crespo-Facorro B, Suárez-Pinilla M. In- and outpatient lifestyle interventions on diet and exercise and their effect on physical and psychological health: a systematic review and meta-analysis of randomised controlled trials in patients with schizophrenia spectrum disorders and first episode of psychosis. Neurosci Biobehav Rev 2021;125:535–68. https://doi.org/10.1016/j.neubiorev.2021.01.005.

[104] Millman LSM, Terhune DB, Hunter ECM, Orgs G. Towards a neurocognitive approach to dance movement therapy for mental health: A systematic review. Clin Psychol Psychother 2021;28:24–38. https://doi.org/10.1002/cpp.2490.

[105] Mössler K, Chen X, Heldal TO, Gold C. Music therapy for people with schizophrenia and schizophrenia-like disorders. Cochrane Database Syst Rev 2011:CD004025. https://doi.org/10.1002/14651858.CD004025.pub3.

[106] Geretsegger M, Mössler KA, Bieleninik Ł, Chen X-J, Heldal TO, Gold C. Music therapy for people with schizophrenia and schizophrenia-like disorders. Cochrane Database Syst Rev 2017;5:CD004025. https://doi.org/10.1002/14651858.CD004025.pub4.

[107] Potes A, Souza G, Nikolitch K, Penheiro R, Moussa Y, Jarvis E, et al. Mindfulness in severe and persistent mental illness: a systematic review. Int J Psychiatry Clin Pract 2018;22:253–61. https://doi.org/10.1080/13651501.2018.1433857.

[108] Rus-Calafell M, Garety P, Sason E, Craig TJK, Valmaggia LR. Virtual reality in the assessment and treatment of psychosis: a systematic review of its utility, acceptability and effectiveness. Psychol Med 2018;48:362–91. https://doi.org/10.1017/S0033291717001945.

[109] van den Noort M, Yeo S, Lim S, Lee S-H, Staudte H, Bosch P. Acupuncture as Add-On Treatment of the Positive, Negative, and Cognitive Symptoms of Patients with Schizophrenia: A Systematic Review. Med Basel Switz 2018;5:E29. https://doi.org/10.3390/medicines5020029.

[110] Roberts MT, Lloyd J, Välimäki M, Ho GW, Freemantle M, Békefi AZ. Video games for people with schizophrenia. Cochrane Database Syst Rev 2021;2:CD012844. https://doi.org/10.1002/14651858.CD012844.pub2.

[111] Brunoni AR, Vanderhasselt M-A. Working memory improvement with non-invasive brain stimulation of the dorsolateral prefrontal cortex: a systematic review and meta-analysis. Brain Cogn 2014;86:1–9. https://doi.org/10.1016/j.bandc.2014.01.008.

[112] Mondino M, Bennabi D, Poulet E, Galvao F, Brunelin J, Haffen E. Can transcranial direct current stimulation (tDCS) alleviate symptoms and improve cognition in psychiatric disorders? World J Biol Psychiatry Off J World Fed Soc Biol Psychiatry 2014;15:261–75. https://doi.org/10.3109/15622975.2013.876514.

[113] Dougall N, Maayan N, Soares-Weiser K, McDermott LM, McIntosh A. Transcranial magnetic stimulation (TMS) for schizophrenia. Cochrane Database Syst Rev 2015:CD006081. https://doi.org/10.1002/14651858.CD006081.pub2.

[114] Wang W, Pu C, Jiang J, Cao X, Wang J, Zhao M, et al. Efficacy and safety of treating patients with refractory schizophrenia with antipsychotic medication and adjunctive electroconvulsive therapy: a systematic review and meta-analysis. Shanghai Arch Psychiatry 2015;27:206–19. https://doi.org/10.11919/j.issn.1002-0829.215093.

[115] Hasan A, Strube W, Palm U, Wobrock T. Repetitive Noninvasive Brain Stimulation to Modulate Cognitive Functions in Schizophrenia: A Systematic Review of Primary and Secondary Outcomes. Schizophr Bull 2016;42 Suppl 1:S95–109. https://doi.org/10.1093/schbul/sbv158.

[116] Kedzior KK, Gierke L, Gellersen HM, Berlim MT. Cognitive functioning and deep transcranial magnetic stimulation (DTMS) in major psychiatric disorders: A systematic review. J Psychiatr Res 2016;75:107–15. https://doi.org/10.1016/j.jpsychires.2015.12.019.

[117] Martin DM, McClintock SM, Forster J, Loo CK. Does Therapeutic Repetitive Transcranial Magnetic Stimulation Cause Cognitive Enhancing Effects in Patients with Neuropsychiatric Conditions? A Systematic Review and Meta-Analysis of Randomised Controlled Trials. Neuropsychol Rev 2016;26:295–309. https://doi.org/10.1007/s11065-016-9325-1.

[118] Zheng W, Cao X-L, Ungvari GS, Xiang Y-Q, Guo T, Liu Z-R, et al. Electroconvulsive Therapy Added to Non-Clozapine Antipsychotic Medication for Treatment Resistant Schizophrenia: Meta-Analysis of Randomized Controlled Trials. PloS One 2016;11:e0156510. https://doi.org/10.1371/journal.pone.0156510.

[119] Ali SA, Mathur N, Malhotra AK, Braga RJ. Electroconvulsive Therapy and Schizophrenia: A Systematic Review. Mol Neuropsychiatry 2019;5:75–83. https://doi.org/10.1159/000497376.

[120] Hauer L, Sellner J, Brigo F, Trinka E, Sebastianelli L, Saltuari L, et al. Effects of Repetitive Transcranial Magnetic Stimulation over Prefrontal Cortex on Attention in Psychiatric Disorders: A Systematic Review. J Clin Med 2019;8:E416. https://doi.org/10.3390/jcm8040416.

[121] Iimori T, Nakajima S, Miyazaki T, Tarumi R, Ogyu K, Wada M, et al. Effectiveness of the prefrontal repetitive transcranial magnetic stimulation on cognitive profiles in depression, schizophrenia, and Alzheimer’s disease: A systematic review. Prog Neuropsychopharmacol Biol Psychiatry 2019;88:31–40. https://doi.org/10.1016/j.pnpbp.2018.06.014.

[122] Jiang Y, Guo Z, Xing G, He L, Peng H, Du F, et al. Effects of High-Frequency Transcranial Magnetic Stimulation for Cognitive Deficit in Schizophrenia: A Meta-Analysis. Front Psychiatry 2019;10:135. https://doi.org/10.3389/fpsyt.2019.00135.

[123] Begemann MJ, Brand BA, Ćurčić-Blake B, Aleman A, Sommer IE. Efficacy of non-invasive brain stimulation on cognitive functioning in brain disorders: a meta-analysis. Psychol Med 2020;50:2465–86. https://doi.org/10.1017/S0033291720003670.

[124] Kostova R, Cecere R, Thut G, Uhlhaas PJ. Targeting cognition in schizophrenia through transcranial direct current stimulation: A systematic review and perspective. Schizophr Res 2020;220:300–10. https://doi.org/10.1016/j.schres.2020.03.002.

[125] Narita Z, Stickley A, DeVylder J, Yokoi Y, Inagawa T, Yamada Y, et al. Effect of multi-session prefrontal transcranial direct current stimulation on cognition in schizophrenia: A systematic review and meta-analysis. Schizophr Res 2020;216:367–73. https://doi.org/10.1016/j.schres.2019.11.011.

[126] Yu L, Fang X, Chen Y, Wang Y, Wang D, Zhang C. Efficacy of transcranial direct current stimulation in ameliorating negative symptoms and cognitive impairments in schizophrenia: A systematic review and meta-analysis. Schizophr Res 2020;224:2–10. https://doi.org/10.1016/j.schres.2020.10.006.

[127] Ciullo V, Spalletta G, Caltagirone C, Banaj N, Vecchio D, Piras F, et al. Transcranial Direct Current Stimulation and Cognition in Neuropsychiatric Disorders: Systematic Review of the Evidence and Future Directions. Neurosci Rev J Bringing Neurobiol Neurol Psychiatry 2021;27:285–309. https://doi.org/10.1177/1073858420936167.

[128] Laskov O, Klírová M. Effects of deep transcranial magnetic stimulation (dTMS) on cognition. Neurosci Lett 2021;755:135906. https://doi.org/10.1016/j.neulet.2021.135906.

[129] Liu Y, Gu N, Cao X, Zhu Y, Wang J, Smith RC, et al. Effects of transcranial electrical stimulation on working memory in patients with schizophrenia: A systematic review and meta-analysis. Psychiatry Res 2021;296:113656. https://doi.org/10.1016/j.psychres.2020.113656.

[130] Sloan NP, Byrne LK, Enticott PG, Lum JAG. Non-Invasive Brain Stimulation Does Not Improve Working Memory in Schizophrenia: A Meta-Analysis of Randomised Controlled Trials. Neuropsychol Rev 2021;31:115–38. https://doi.org/10.1007/s11065-020-09454-4.

[131] Sun C-H, Jiang W-L, Cai D-B, Wang Z-M, Sim K, Ungvari GS, et al. Adjunctive multi-session transcranial direct current stimulation for neurocognitive dysfunction in schizophrenia: A meta-analysis. Asian J Psychiatry 2021;66:102887. https://doi.org/10.1016/j.ajp.2021.102887.

[132] Yamada Y, Inagawa T, Hirabayashi N, Sumiyoshi T. Emotion Recognition Deficits in Psychiatric Disorders as a Target of Non-invasive Neuromodulation: A Systematic Review. Clin EEG Neurosci 2021:1550059421991688. https://doi.org/10.1177/1550059421991688.
